# Diversity, expression and mRNA targeting abilities of Argonaute-targeting miRNAs among selected vascular plants

**DOI:** 10.1186/1471-2164-15-1049

**Published:** 2014-12-02

**Authors:** Soham Jagtap, Padubidri V Shivaprasad

**Affiliations:** National Centre for Biological Sciences, GKVK Campus, Bellary Road, Bangalore, 560 065 India

**Keywords:** Plant miRNAs, Plant silencing, Argonaute, Dicer-like, miR168, miR403

## Abstract

**Background:**

Micro (mi)RNAs are important regulators of plant development. Across plant lineages, Dicer-like 1 (DCL1) proteins process long ds-like structures to produce micro (mi) RNA duplexes in a stepwise manner. These miRNAs are incorporated into Argonaute (AGO) proteins and influence expression of RNAs that have sequence complementarity with miRNAs. Expression levels of AGOs are greatly regulated by plants in order to minimize unwarranted perturbations using miRNAs to target mRNAs coding for AGOs. AGOs may also have high promoter specificity-sometimes expression of AGO can be limited to just a few cells in a plant. Viral pathogens utilize various means to counter antiviral roles of AGOs including hijacking the host encoded miRNAs to target AGOs. Two host encoded miRNAs namely miR168 and miR403 that target AGOs have been described in the model plant *Arabidopsis* and such a mechanism is thought to be well conserved across plants because AGO sequences are well conserved.

**Results:**

We show that the interaction between AGO mRNAs and miRNAs is species-specific due to the diversity in sequences of two miRNAs that target AGOs, sequence diversity among corresponding target regions in AGO mRNAs and variable expression levels of these miRNAs among vascular plants. We used miRNA sequences from 68 plant species representing 31 plant families for this analysis. Sequences of miR168 and miR403 are not conserved among plant lineages, but surprisingly they differ drastically in their sequence diversity and expression levels even among closely related plants. Variation in miR168 expression among plants correlates well with secondary structures/length of loop sequences of their precursors.

**Conclusions:**

Our data indicates a complex AGO targeting interaction among plant lineages due to miRNA sequence diversity and sequences of miRNA targeting regions among AGO mRNAs, thus leading to the assumption that the perturbations by viruses that use host miRNAs to target antiviral AGOs can only be species-specific. We also show that rapid evolution and likely loss of expression of miR168 isoforms in tobacco is related to the insertion of MITE-like transposons between miRNA and miRNA* sequences, a possible mechanism showing how miRNAs are lost in few plant lineages even though other close relatives have abundantly expressing miRNAs.

**Electronic supplementary material:**

The online version of this article (doi:10.1186/1471-2164-15-1049) contains supplementary material, which is available to authorized users.

## Background

Plant miRNAs are indispensable for the control of wide variety of biological functions, including development, hormone responses, feedback mechanisms and biotic and abiotic stresses [1, 2]. Most of these functions are associated with ability of miRNAs in targeting mRNAs coding for transcription factors and other key genes [3]. Nearly half of all known plant miRNAs that are highly conserved across plants target transcription factors [4], justifying their importance in the regulation of plant processes. The remaining half of less-conserved miRNAs regulate expression of a variety of protein coding genes involved in metabolic processes, transporters and the process of RNA silencing itself [4]. Additionally, role of some less-conserved miRNAs in controlling disease resistance in a variety of plants has been recently reported [5–7]. The well-conserved miRNAs that are evolutionarily ancient have many copies in the genomes, sometimes up to 50 copies, largely due to genome duplications or due to the rapid evolution of their target mRNAs, or both [3, 4, 8]. The duplicated copies called ‘isoforms’ may have high similarity in their mature miRNA sequences, but the similarity is less obvious among regions beyond miRNA stem-loops [9–11]. The less conserved miRNAs usually have 1 or 2 copies of themselves with high sequence similarity throughout the length of their RNAs [3, 4, 8, 12].

miRNAs are generated in a stepwise manner from long non-coding, genome-encoded PolII transcripts. Primary miRNA transcripts of varied length must form a secondary structure that can be recognized by Dicer-like protein 1 (DCL1) and its partners. DCL1 complex cleaves the secondary structured RNA to a pre-miRNA structure having a stem with almost complete complementarity and a loop. Another processing step in the cytoplasm produces miRNA:miRNA* duplex of predominantly 21 nts with 2 nt overhangs. While miRNA* is usually degraded, miRNAs associate with Argonaute (AGO) proteins to form RNA-induced silencing complexes (RISC). Most of the plant miRNAs appear to be targeting mRNAs for degradation, typically ‘slicing’ target mRNA between position 10 and 11. There are also many reports of plant miRNA:mRNA complexes leading to translational inhibition [1, 2].

Many factors in miRNA pathway have been characterized, but there is limited information on the self-regulation of miRNA pathway itself. Three miRNAs have been reported to regulate DCL and AGO by cleaving transcripts of corresponding targets as part of a robust feedback mechanism. miR162 has been implicated in targeting DCL1 [13], miR168 in AGO1 mRNA [14–16] and miR403 in targeting AGO2 and AGO3 mRNAs in *Arabidopsis* and other related plants [17–19]. The regulation of AGOs is quite striking because miRNAs that target mRNAs of AGOs need to form RISC complexes with AGO proteins themselves.

A relatively well-known feedback mechanism involves AGO1 homeostasis that is controlled by coordinated action of miR168 [15, 20] and AGO1-derived siRNAs [21] on AGO1 mRNA. Vaucheret et al. [22] also identified an additional complexity of this interaction involving the AGO1-mediated post-transcriptional stabilization of miR168 and the co-regulated expression of AGO1 and miR168 genes in *Arabidopsis*. Thus, it appears that *Arabidopsis* has a refined feedback regulatory loop that balances AGO1 and miR168 accumulation. In addition, miR168 expression is regulated by invading viruses. Upon infection with wide range of viruses, miR168 levels go up to dramatic levels quite quickly, leading to the repression of AGO1 translation [16]. A similar upregulation of miR403, though proposed [17], has not been experimentally verified. Regulation of AGOs by miR168 and miR403 has been proposed to be conserved among many plants in addition to tobacco and *Arabidopsis*, although an in-depth analysis is not forthcoming.

The induction of miRNAs by viruses to meddle with AGO expression indicates that pathogens use these miRNAs particularly to suppress host silencing. This implies that variation in expression levels or targeting abilities of miRNAs among plant lineages has the potential to be the basis of susceptibility or resistance against pathogens. In order to understand these relationships, we analyzed sequence diversity, copy numbers and expression levels of miR168 and miR403 among plant species for which a small RNA dataset is available. Surprisingly, our results suggest that mature sequences of miR168 can be classified into three clades. Presence of three clades is also evident after aligning precursor sequences of these miRNAs. Variation in miR168 sequences seems to correlate well with their AGO1 mRNA targeting abilities. Strikingly, tobacco (*N. tabacum*) has isoforms with insertion of transposon-like sequences that are likely to reduce their processing, providing a clue to the loss of miRNAs. Furthermore, when we analyzed miR168 sequences across 68 plant species representing almost all major plant families, a clade specific abundance of miR168 was observed. The maximum abundance and diversity of miR168 was among monocots. Monocots have more numbers of AGO1 members than dicots, but few of them are not readily targeted by miR168 creating functional difference. The regulation of AGO2 and AGO3 mRNAs by miR403 is specific to very few plants. miR403 is absent among monocots and many eudicot lineages. Absence of miR403 among monocots indicates an inverse relationship with that of miR168, since miR168 is highly expressed among all monocots studied. In those plants where miR403 is present, the sequences are less diverse and plants have fewer isoforms of this miRNA indicating recent evolution of miR403. Together, this analysis indicates that miR168/miR403 relationships with their targets as observed in *Arabidopsis* are likely to be specific only to few plant lineages and plants have evolved every shade of such regulation providing case for altered transcription factor regulations and disease resistance.

## Results

### miR168 sequences from diverse plant families fall into three distinct clades

**Figure 1:**
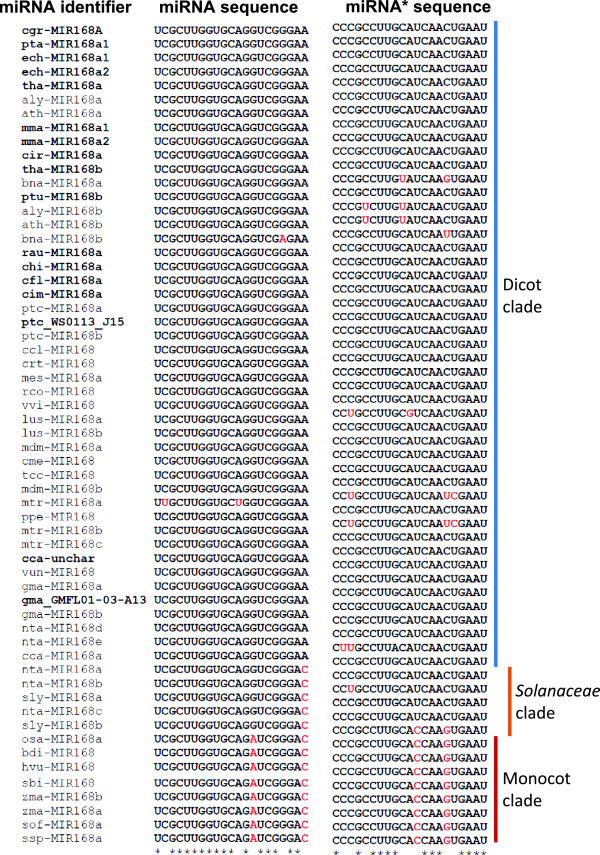
Multiple sequence alignment of miR168 and miR168* sequences from vascular plants. Sequences from miRBase as well those fetched from other sources (in bold, see materials and methods) were aligned using ClustalW. Residues in red are not conserved among others. Expanded names of species that are abbreviated are given in Additional file 1: Table S1.

In order to understand the sequence diversity of miR168, we used sequences from miRBase (version 20) as well as from genome-wide transcriptome data reported from plants that have been studied. A total of 58 sequences were obtained representing 31 families of plants. Among these, 16 were newly designated sequences. All 58 miR168 and miR168* sequences were used for sequence alignment (Figure 1, Additional file 1: Table S1) that shows diversity in mature miRNA sequences. Similar miR168 diversity has been documented by a comprehensive analysis reported recently [12]. The mature miR168 sequences can be classified into 3 groups, a large dicot group representing most of the reported miR168 sequences, a monocot-specific group with sequence variations at positions 14 and 21 and a third group of miRNAs with intermediate sequence variation was observed among *Solanaceae* members. *Solanaceae* members exhibited similar sequence in 14^th^ position (G) like other dicots, but had similarity at 21^st^ position (C) identical to monocots. The miR168* sequences, on the other hand, had uniform sequence diversity among monocots. Distinct miR168* sequences for *Solanaceae* members were not observed. Among *Solanaceae* members, *Nicotiana tabacum* showed an unusual sequence diversity with two forms (d, e) showing mature miRNA sequences similar to other dicots while three additional isoforms (a,b,c) having a *Solanaceae* specific sequence signature. Two sequences from the dicot clade, miR168 from *Brassica napus* and *Medicago truncatula,* had variations that could not be compared to three clades and are likely results of rapid independent evolution.

In order to verify the presence of three groups of miR168 sequences, we aligned the precursors of all miR168 sequences using ClustalW2. Phylogenetic tree derived from this alignment (Additional file 2: Figure S1) also confirms the presence of three clades of miR168 sequences. This suggests that there are sequences beyond mature miRNA sequences that also contribute towards clade-specificity. Presence of these clades and a separate clade for *Solanaceae* is remarkable because this indicates independent evolution of miR168 among *Solanaceae* members as these species are distantly related to monocots.

**Figure 2:**
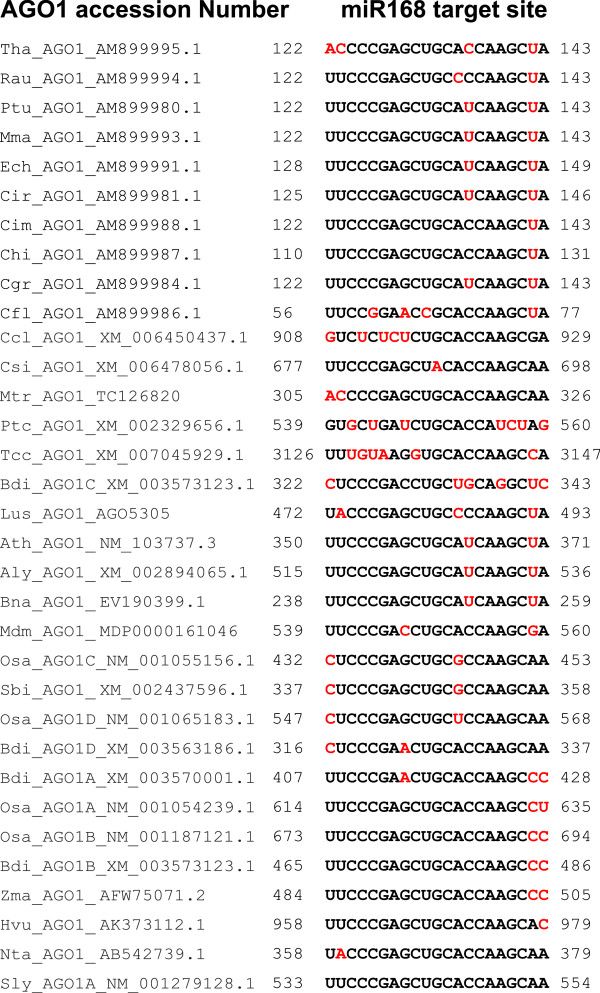
**Alignment of miR168 targeting regions in AGO1 from various plant species.** Residues in red are not conserved among others. Start and stop regions in AGO1 mRNAs have been mentioned.

If the miRNAs and their targets co-evolved with their target genes in different plant lineages as proposed [4, 23, 24], then the target mRNA regions of these miRNAs must have clade-specific changes. However, among the sequences of AGO1 mRNAs from corresponding plant species, there are hardly any clade/family specific changes in the miR168 target regions (Figure 2). The miR168 target region in AGO1 mRNAs does not code for a key RNA motif that will code for a conserved domain, however, there is still high sequence conservation among AGO1 sequences derived from distinct species. This also indicates evolutionarily ancient interaction between miR168 and AGO1. A slightly higher AGO1 sequence divergence in the miRNA target region was observed among phylogenetically unrelated species such as *Populus trichocarpa* (*Salicaceae*), *Cardamina flexuosa* (*Brassicaceae*), *Citrus clementine* (*Rutaceae*), *Theobroma cacao* (*Malvaceae*) and *Brachypodium distachyon* (*Poaceae*), functional significance of which is unknown.

**Figure 3:**
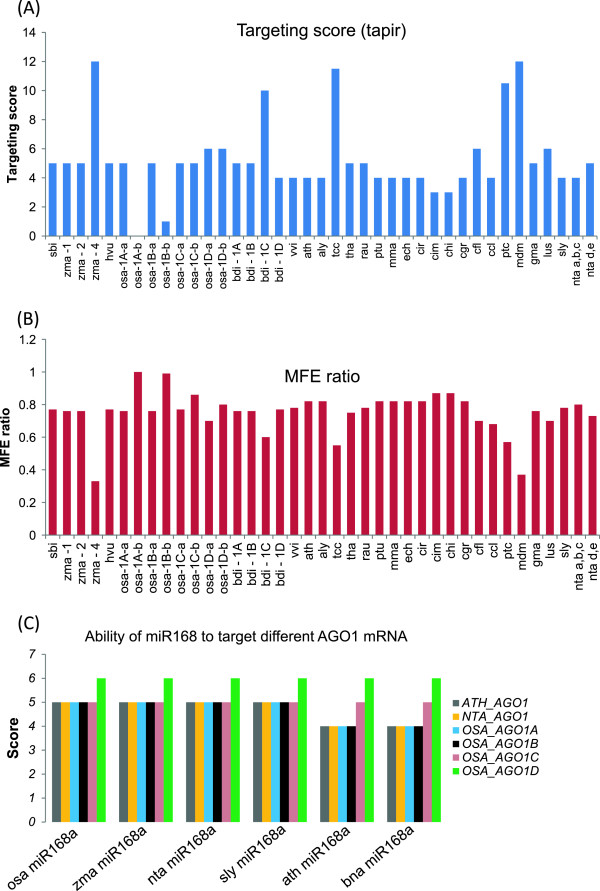
**AGO1 targeting abilities of miR168 from corresponding species. (A)** Target score for AGO1 targeting in different species by their miR168. TAPIR analysis was carried out as described in methods section. Abbreviation of plant names are given in Additional file 1: Table S1. **(B)** MFe ratios for the target/miR168 complementarity. Best targets have lower score and higher MFe ratio. **(C)** Predicted AGO1 targeting of miR168 from few representative species along with AGO1 from other species for comparison.

It is unclear whether miRNAs from one plant species target their mRNAs to the same levels as in other species as such studies have not been carried out. In order to understand the significance of variations in miR168 mature sequences we predicted the miR168 targeting abilities among AGO1 mRNAs of all plant species that have clearly identified miR168 sequences. We used both pssRNAtarget [25] as well tapir [26], two tools that have been acknowledged to provide high reliability [27]. Figure 3 indicates uniformly high targeting (5 or lower tapir score) for most AGO1 mRNAs irrespective of being monocots or dicots, while few showed almost no targeting. *Populus trichocarpa*, *Malus domestica*, *Theobroma cacao* showed very high tapir scores of 10.5, 12 and 7, respectively, and correspondingly low MFe ratios indicating that their AGO1 mRNAs may not be targets of their miR168.

Monocots show higher diversification of AGO1s, usually with 4 members [14], something that may have been due to ancient duplications [28]. We anticipated that presence of additional AGO1 mRNA may provide differential targeting abilities for monocot miR168 members (Additional file 3: Figure S2). Although there are 4 members of AGO1 there are only one or two distinct mature miR168 sequences. Some AGO1 members of *Zea mays* such as AGO1c and AGO1d are not targeted by zma miR168, but AGO1a and b are good targets (Additional file 3: Figure S2). Rice AGO1d alone has slightly higher tapir score (less optimal target due to mismatch in cleavage site or nearby), while other AGO1 members are good targets. Among the 4 AGO1 members from *Brachypodium*, one member (Bdi AGO1c) is clearly not a target of bdi miR168 (more than 3 mismatches in seed region). These differences among monocots might contribute to the diversity in their miRNA pathways since AGO1 is a major player in miRNA stabilization and action. We also predicted targets for few miR168 examples from each of the three clades with AGO1 mRNAs from the corresponding plants (Figure 3C). This analysis shows that targeting of AGO1 mRNAs by miR168 in monocots is slightly less intense than among dicots based on tapir score.

### Secondary structures and precursor miRNA features of miR168s indicate their rapid diversification in *Solanaceae*clade

**Figure 4:**
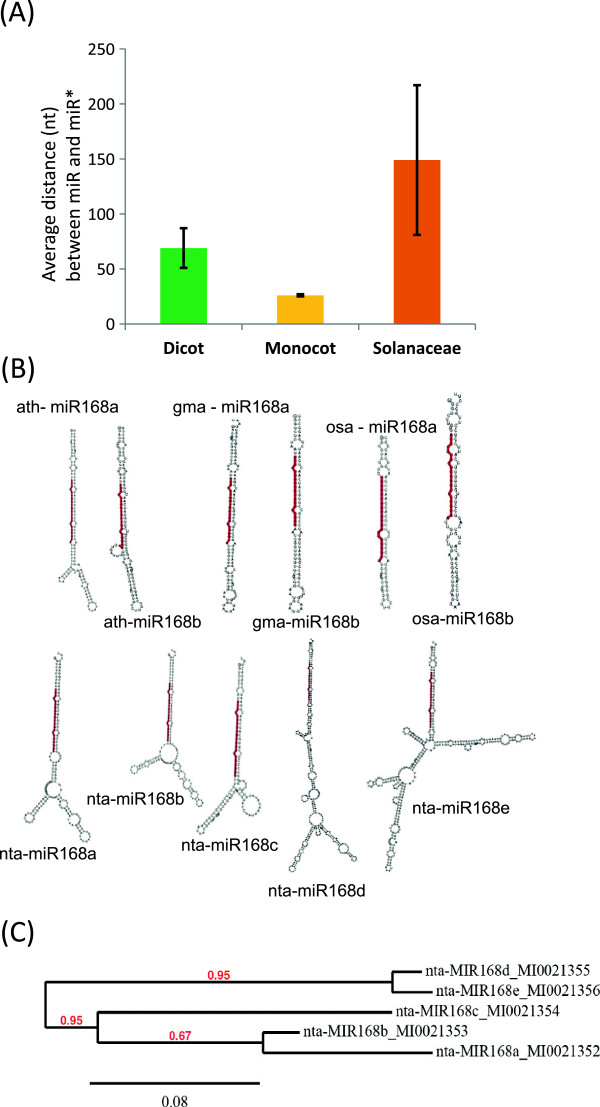
**Structural variations in tobacco miR168 isoforms compared to other representative plants. (A)** Variation in average length between miR168 and miR168* sequences among monocots, all dicots except *Solanaceae* and among *Solanaceae*. **(B)** Secondary structures of miR168 members from few plants. Although rice, soybean and *Arabidopsis* have 2 identical mature miR168 isoforms with almost similar secondary structures, tobacco isoforms have diverse secondary structures. RNA fold (http://rna.tbi.univie.ac.at/cgi-bin/RNAfold.cgi) was used to determine secondary structures. **(C)** Phylogenetic analysis of miR168 isoforms from tobacco indicating two clusters. Tree was constructed as described in methods section.

The sequence and distance between miRNAs and miRNA* must indicate evolutionary history of miRNAs [29]. However, these sequences that make up the ‘loop’ are critical for host DCL1 to process the long non-coding RNAs into short miRNAs duplexes [30]. The distance between miR and miR* were quite similar among most dicot miR168 precursors, ranging typically between 50 and 80 nts (Additional file 4: Figure S3). Surprisingly, monocots had very short loop sequences in the range of 20–30 (Figure 4A). Among the *Solanaceae* members there were two distinct groups. One group of precursor sequences that make mature miRNAs common to other dicots, have length of loop sequences between 70–90 typical of other dicots, while some members (nta miR168d and nta miR168e) have unusually long (up to 290 nt) loops.

**Figure 5:**
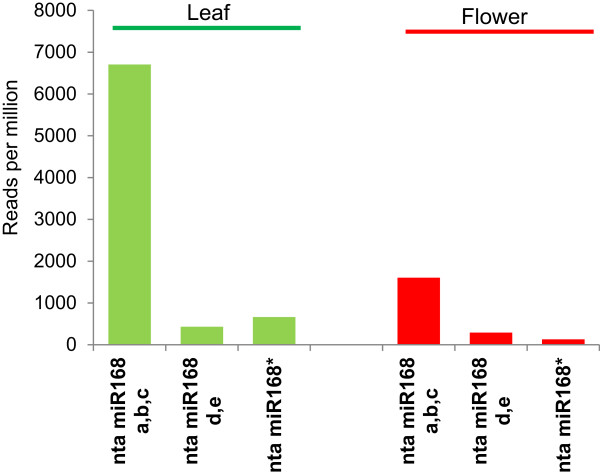
**Accumulation of miR168 a,b,c and miR168 d,e isoforms among tobacco tissues (floral and leaf).** Reads of miRNA or miRNA* per million reads was taken from GEO accession GSE28977. Similar ratio between a,b,c and d,e were observed among tobacco pods. miR168 sequences were retrieved as discussed in methods section.

Because structures of these miRNA precursors are crucial for their biogenesis, a secondary structure prediction of all miR168 precursors was carried out using RNA-fold (Figure 4B, Additional file 5: Figure S4). Among the dicots, the typical secondary structure had one or two small loops and short branches. Monocots having very short loops had a simple stem loop with high sequence complementarity. Usually high sequence complementarity beyond miR and miR* sequences indicate their recent evolution. Among the *Solanaceae*, those with shorter loops had structures similar to other dicots, but as expected the structures of miR168 isoforms with long loops were complex. The nta miR168d and e precursors have long loop sequences similar to each other but extremely different from any other miR168 precursor (Figure 4C). A closer look showed high sequence similarity between fragments of loop sequences between these two precursors and *M*iniature *I*nverted repeat *T*ransposable *E*lements (MITE) from few dicots (Additional file 6: Figure S5). MITEs are cut and paste type transposon elements typically leaving short fragments when they jump to newer locations. The presence of MITE-like sequences in the loop region for any miRNA has not been reported so far. It is important to note that Piriyapongsa et al. [31] have proposed that miRNAs encoded by MITEs evolved from corresponding ancestral full-length (autonomous) elements that originally encoded short interfering (si)RNAs. For miR168 though this may have been in a reverse order. A systematic search using published genomes identified other regions that could have been miR168 precursors that invited other repeat elements to become transcriptionally inactive (data not shown).In tobacco, identification of mature miRNAs corresponding to isoforms a, b, c (without MITE insertion) and d, e (with MITE insertion) is possible due to the sequence divergence between these isoforms. We hypothesized that insertion of MITEs might interfere with Pri-miR168 transcription or biogenesis steps and therefore those isoforms with insertion should be less abundant compared to their counterparts. Strikingly, tobacco miR168d,e isoforms were ~15 times low abundant than a,b,c isoforms in leaves and flowers (Figure 5), supporting the idea that long loop-containing precursors of miR168 yield less abundant mature miRNAs.

### Comparative abundance of miR168 among plant families

**Figure 6:**
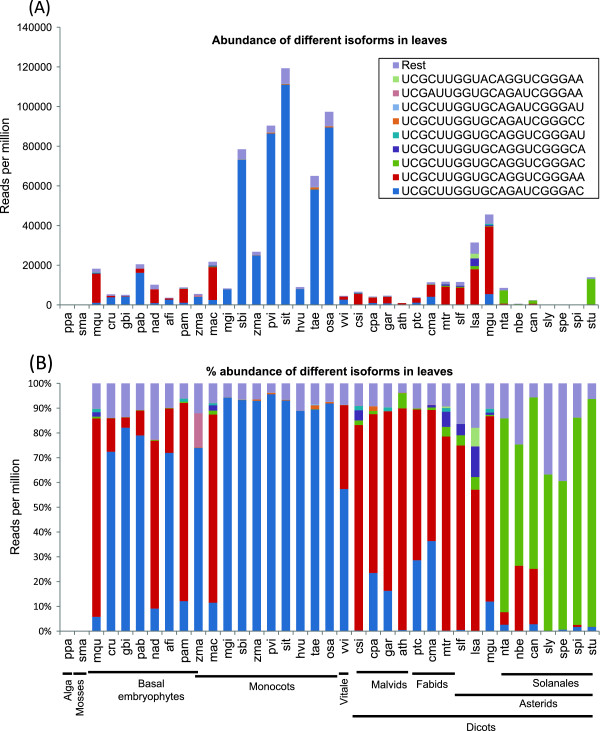
**Abundance and sequence diversity of miR168 members across plant families. (A)** Highest abundance of miR168 among monocots. Color bars represent most abundant forms of miR168 in leaf tissues. Blue, red and green bars represent monocot, dicot and *Solanaceae*-specific forms as shown in Figure 1. Abundance was measured as discussed in methods section. **(B)** Percentage abundance of miR168 across plant families. Phylogenetic relationships among plant species have been indicated.

We used small RNA datasets derived from 42 plant species representing 25 plant families to compare expression and diversity of miR168 sequences. Next generation small RNA sequence datasets from the corresponding species were taken from GEO and other published sources (See materials and methods). The abundance of miR168 and those reads that match to miR168 with 1 or 2 mismatches were taken into consideration (‘miRProf’ tool, [32]) as genomes of many of these plants and information about their miRNAs are not readily available. The data presented in Figure 6A indicates that there is very high expression of miR168 sequences among most monocot families when compared to dicots. The most abundant form of miR168 among *Poaceae* was the monocot-specific form of miR168 (Figure 1, Figure 6B). Similar observations were made in a recent study that compared miRNA diversity across vascular plants [12]. The other major monocot order *Zingiberales* (*Musa acuminata*) on the other hand, seem to have the common dicot specific form as the most abundant. *Musa* not only has a dicot specific form as most abundant form, but also has accumulation of miR168 levels matching those of dicots in that it has low accumulation. The monocot specific form surprisingly is found also among Cycads (*Cycas rumphii*), Gingkophyta (*Gingko biloba*) and Pinophyta (*Picea abies*). These species represent forms that are ancient to monocot/dicot divergence and it is easy to speculate that these two forms are ancient. Magnoliids (*Aristolochia* and *Persea*) show abundance of either mature miR168 form depending on the species. Depending on the plant lineage some miR168 forms could have evolved and expressed better than the other forms. *Vitis vinifera* (*Vitales*), a eudicot, has higher expression of monocot specific form unlike other eudicots for those a sequence information is available, is an example wherein both forms co-exist.

Surprisingly, abundance of miR168 was comparatively low among *Arabidopsis* and members of *Solanaceae* such as *Solanum spp.* and *Nicotiana spp.* Most abundant form of miR168 among *Solanaceae* was the form that is distinct from other dicots. These forms have also been reported recently in a global study using small RNA datasets from many vascular plants [12]. Among dicots, some Astrids such as *Mimulus guttatus* (*Lamiaceae*), *Lactuca sativa* (*Asterales*) have the highest accumulation of miR168.

Consistent with the observation in *Arabidopsis* that floral tissues accumulate more 24 nt siRNAs than miRNAs, miR168 levels are generally low among floral samples of all plant species analyzed. Unexpectedly, rice (*Oryza sativa*) and sorghum (*Sorghum bicolor*) are some of the few species where miR168 was consistently expressed highly among both leaf and floral tissues (Additional file 7: Figure S6).

Viruses as part of their counter-defense strategy target the host AGOs by destabilizing them directly at the protein level [33–36]. Some viruses even act at a higher level by inducing degradation of mRNAs of AGO family members that are part of the host defense, namely AGO1, AGO2 and AGO3. Viral counter-defense by targeting of AGO mRNAs is through induction of miR168 as observed in *N. benthamiana* [16] *, A. thaliana* [22] and *S. lycopersicum* [16]. After analyzing the global miR168 levels in these species it makes sense to hypothesize why viruses need to upregulate miR168 to target AGO1 mRNAs. All these species have very low levels of miR168 in uninfected tissues indicating they may have higher accumulation of AGO1 protein thereby indicating AGO1 as the most likely and potent candidate to target viruses. However, such an interaction seems unlikely for monocots (these plants act as hosts to few viruses), members of which accumulate miR168 at higher levels even without any biotic stress. By analyzing publicly available small RNA datasets we can show that miR168 induction upon virus infection of rice (a monocot) is hardly noteworthy ([37] Additional file 8: Figure S7). Corresponding levels of increase in miR168 levels upon viral infection for few dicots such as *N. benthamiana*, *Arabidopsis* and tomato are at much higher levels [16]. This difference in miR168 levels upon virus infection among dicots and monocots is important because AGO1 protein levels go down dramatically upon virus infection in *Arabidopsis* and *Nicotiana* members [16], while in rice, the reduction in AGO1 protein level is negligible [38]. These results suggest a functional difference in downstream activities of AGO1 among monocots and dicots that is brought about by variation in expression levels of miR168.

### miR403 has low sequence diversity and are present only among selected lineages of plants

**Figure 7:**
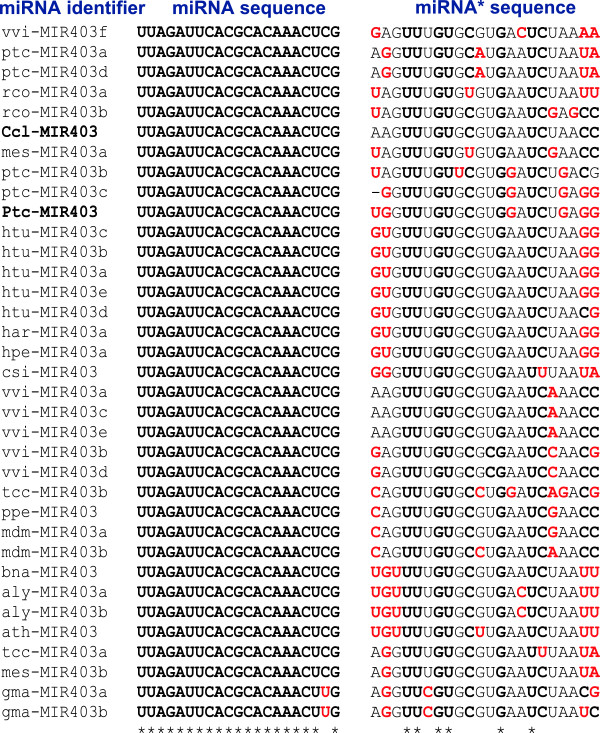
**Diversity of miR403 sequences.** CLUSTAL alignment with sequences from miRBase as well as from other sources (in bold). Residues in red are not conserved among the family members.

A similar search for miR403 in miRBase as well as transcript databases recovered 35 sequences (Figure 7). miR403 has been reported to be absent in monocots [4, 39], but a detailed information of plant families where this miRNA is present is not known. Among the plants where miR403 is reported, *Glycine max* alone has a sequence variation (at position 20) when compared to all other species. This lack of diversity in sequences arises from its recent evolution as this miRNA is present only in few lineages of eudicots. Some plant species have many copies of miR403, for example *Vitis* has 5 copies, all with the same mature sequence but with diverse miR* sequences. The most common and abundant form of the miR403 is 5’-UUAGAUUCACGCACAAACUCG-3’. Few interesting deviations from this sequence were observed in *Solanaceae* members (*S. pennellii* and *S. tubersum*) and some Malvids (*Carica papaya*) sharing 5’-CUAGAUUCACGCACAAACUCG-3’ as the major miR403 isoform. This isoform is extremely interesting in that it has an unusual 5’ terminal nucleotide as C. One more abundant form specific to *Gossypium arboreum* is 5’-UUAGAUUCACGCACAAACUCA-3’, thus, Malvids seem to have the most diversity in miR403 sequences.

**Figure 8:**
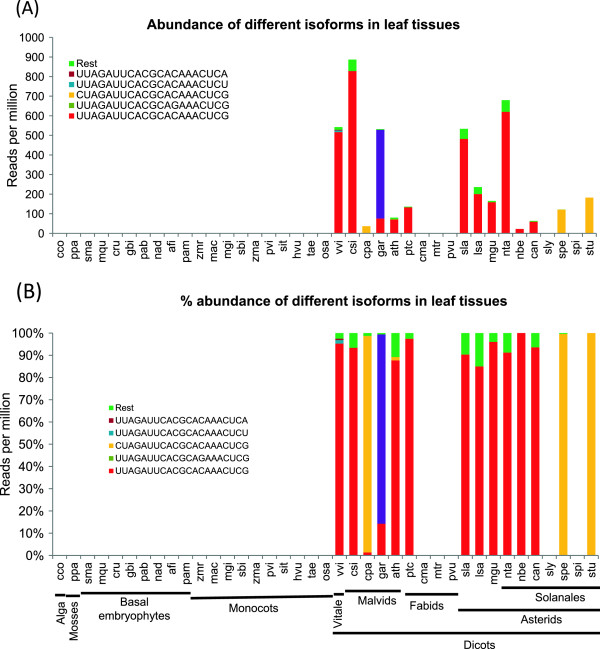
**Abundance and sequence diversity of miR403 members across plant families. (A)** miR403 is conserved only among Rosiid members namely Malvids and Vitales and few Fabids. Color bars represent most abundant forms of miR403 in leaf tissues. Abundance was measured as discussed in methods section. **(B)** Percentage abundance of miR403 across plant families.

Surprisingly, while two sets of Rosiids called Malvids and Vitales have abundant miR403, some Fabids (*Cucurbita, Phaseolus* and *Medicago* spp.) have no expression of miR403 (Figure 8). However, *Populus trichocarpa* (*Salicaceae*), a member of Malphigiales that belong to *Fabidae* have high expression of miR403. Vitales have very high expression both in vegetative and reproductive tissues (Additional file 9: Figure S8), followed by few *Solanaceae* members (*Solanum* and *Nicotiana* spp.).

### Targeting of AGO2 and AGO3 mRNAs by miR403 is restricted to few plant lineages

miR403 targets mRNAs of AGO2 and AGO3 in Arabidopsis. It has been proposed that viruses may interfere with miR403 expression in order to reduce the expression of AGO2, considered as a major antiviral AGO. When we analyzed AGO2 mRNAs from those species where miR403 accumulation could be detected, it was observed that targeting of AGO2 mRNA is very efficient in *Brassicaceae* (*Brassica* and *Arabidopsis* Spp.) and Vitales (*Vitis vinifera*, *Citrus sinensis*), while it is ineffective with score of excess of 10 among Fabids (Additional file 10: Figure S9A and B). It is important to note that *Vitales* have not only very high expression of miR403, but also have high AGO2 targeting ability compared to other plant families. We also checked targeting of AGO3 mRNAs by miR403. Such a targeting seem to be possible only among *Arabidopsis* Spp. (Additional file 11: Figure S10) but not among members of any other plant family. Functional significance of this relationship is not known.

## Discussion

### AGO expression levels and susceptibility to viruses

It has been clearly shown that few AGOs in addition to being important for development by controlling transcription factors and other endogenes, are the first line of defense against incoming pathogens. Two lines of evidence are strongly in favor of this observation. Firstly, viral and other pathogen-derived siRNAs are incorporated into AGO1 and to less extent AGO2, thereby performing the role of targeting complementary mRNAs derived from the incoming pathogens themselves. Secondly, pathogens have the ability to selectively target these AGOs both at post-transcriptional as well as post-translational steps. Since AGO1 is responsible for stabilization and activity of most miRNAs, it has been well documented that miRNA levels change generally during pathogenesis, especially during virus infections, due to AGO1 targeting. Unlike most miRNAs that change marginally during viral infections, levels of miR168 changes drastically. This specific change is likely brought about by viral counter-defense proteins, but the mechanism is largely unknown. However, it has been clearly shown that this interaction is crucial for pathogenicity. Thus, absence or lower expression of miR168 in some plants (Figure 6) may indicate a higher expression of AGO1 in those species and this is where viruses might need to target AGO1 as part of their counter defense strategy. In line with this hypothesis, virus-mediated induction of miR168 has been observed among those plants where miR168 levels are generally lower in the absence of viral infections. On the other hand, higher expression of miR168 in some plant lineages might be resulting in a lower accumulation of corresponding AGOs. Indeed in leaves and roots of rice, a monocot, where miR168 levels are quite high, expression of AGO1s is comparatively at lower levels than in *Arabidopsis* [40, 41]. In such cases, viruses need not target AGO1 as part of their counter-defense simply because antiviral role by AGO1 may be negligible or is taken over by other AGOs. This may be the case for rice, where neither a strong induction of miR168, nor a corresponding reduction in AGO1 levels has been observed upon viral infections (Additional file 8: Figure S7, [38]).

On the other hand, absence of miR403 among monocots correlates well with the expression levels of AGO2. In rice, AGO2 expression is very high among all tissues unlike in Arabidopsis where AGO2 expression is largely confined to siliques and at much lower levels in leaves [40, 41]. It remains to be investigated if AGO2 indeed acts as an antiviral AGO among monocots. However, unlike in *Arabidopsis* where AGO2 gets induced during viral infections [17], there is hardly any change in AGO2 levels during *Rice stripe virus* and *Rice dwarf virus* infections in rice [38]. This supports our view that induction and regulation of AGOs by viruses is restricted to few plant lineages.

### Rapid evolution of tobacco miR168 isoforms

A case for loss of miRNAs can be argued based on the miR168 diversity in *N. tabacum*. The common dicot forms of miR168 in tobacco (isoforms d and e) have insertions of MITE-like transposons. A direct result of this insertion corresponds to reduced accumulation of mature miRNA forms in all tissue types. The *Solanaceae*-specific forms on the other hand (a, b and c), does not have MITE-like insertions and are expressed at high levels. A duplication event of miR168 in tobacco might have ended up with isoforms having two different miR168 sequences. One set (d and e) while retaining mature miR168 of dicot ancestor, attracted transposons in the loop region. Tobacco seems to be a hotbed for miR168 duplication since it already has the most diverse miR168 precursors. In addition, there are many tobacco genomic regions matching miR168 sequences present in the genome, sometimes their miR* sequences either missing or present a long distance downstream or upstream. These may represent the lost miR168 isoforms. It is possible that miR168 example that is observed in tobacco is seen in other miRNAs where some forms are lost during duplication and subsequent loss.

Transposons invite siRNAs and methylation. Invasive Transposons such as MITES can be detected and neutralized using RNA-directed DNA methylation [42]. This may be how promoters of some miRNAs become methylated and inactive. It is possible thus that tobacco miR168 d and e isoforms may have been targets of RNA-directed DNA methylation and hence no longer express their RNAs. It will be interesting to speculate if viruses can still modulate expression of tobacco miR168d and e. Unfortunately, there are no small RNA datasets from tobacco infected with viruses to see if miR168 d and e isoforms are induced upon virus infection. However, other line of evidence suggests that these isoforms are not likely induced upon virus infections. Usually upon virus infections, not just mature miR168, but also its stem-loop structures over-accumulate [16]. However, miR168d and e may not be induced by viruses as seen from the absence of accumulation of their precursors or pre-miRNAs [16]. A stem-loop of miR168 d and e (they are around 200 + nts) was not seen among tobacco samples infected with viruses.

## Conclusions

Our study indicates that intricacies of AGO targeting by miRNAs as observed in *Arabidopsis* is specific only among few plant lineages. Plants have evolved every shade of this regulation providing case for varying miRNA levels, thus influencing transcription factor and other activities regulated by miRNAs. The nature of this interaction may also influence disease resistance due to the way viruses use these miRNAs to manage the arms race with their host plants.

## Methods

### Identifying miRNA Diversity, Sequence Alignments and their Targets

The UEA small RNA analysis toolkit [32] was used to identify members of a given miRNA family (miRProf and miRCat) using default as well as allowing 3 mismatches. Detailed description of the tool is given in http://srna-tools.cmp.uea.ac.uk/. Sequences of miR168 and miR403 were obtained from miRBase release 20 [43, 44] and aligned using ClustalW and ClustalX2 [45]. Previously unreported sequences of miR168 and miR403 were obtained from EST datasets after fulfilling criteria for plant miRNAs including checking for secondary structure (RNAfold, [32]) as well as abundance and distribution of small RNAs across the length of the precursors. List of all plants species and their families are given in Additional file 1: Table S1. Targets of miRNA were identified using two different algorithms, namely, psRNATarget algorithm [25] and TAPIR algorithm [26]. To find targets of miR168 and miR403 family in the plant genomes, target AGO sequences were taken from the NCBI [46], Solanaceous genome Network [47], Refseq [48] as well as from NCBI GEO [49].

Analysis of abundance of miR168 and miR403 members were performed through miRProf analysis of published large-scale data sets derived from various plant species available through the Gene Expression Omnibus (GEO) platform [49, 50]. These libraries have been described previously [5, 6, 12, 30, 51–57]. miRNA sequences were checked to compensate for the mis annotation of miR168 type and miR403-type sequences in miRBase.

### Phylogenetic analysis

The phylogenetic tree was constructed using the MEGA 6.0 software with Neighbor Joining Method with 1000 bootstrap replications. The model used was Jukes Cantor that had the highest log-likelihood score according to the J-model Test (https://code.google.com/p/jmodeltest2/). For the J-model test the precursor alignment was given as the input in “.aln” format. It calculates for the variations in the nucleotide sequences and gives the log-likelihood scores for all the models for phylogenetic tree construction.

## Electronic supplementary material

Additional file 1: Table S1: Plant species used for small RNA analysis. (PPTX 68 KB)

Additional file 2: Figure S1: Phylogenetic analysis of precursors of miR168. (PPTX 62 KB)

Additional file 3: Figure S2: Targeting abilities of miR168 among selected plants indicating mosaic targeting among multiple AGO1 members in monocots. (PPTX 675 KB)

Additional file 4: Figure S3: Length of loops (distance between miRNA and miRNA*) among 58 miR168 precursors from diverse plants. (PPTX 94 KB)

Additional file 5: Figure S4: Secondary structures of 58 precursors of miR168 indicating clade-specific changes in the loop region. (PPTX 620 KB)

Additional file 6: Figure S5: Multiple sequence alignment of miR168 precursors from *Solanaceae* indicating site of MITE insertion. (PPTX 79 KB)

Additional file 7: Figure S6: Abundance and sequence diversity of miR168 members across plant families in reproductive tissues. (PPTX 88 KB)

Additional file 8: Figure S7: miR168 is not significantly induced in rice upon infection with viruses. (PPTX 63 KB)

Additional file 9: Figure S8: Abundance and sequence diversity of miR403 members across plant families in reproductive tissues. (PPTX 6 MB)

Additional file 10: Figure S9: Ago2 targeting abilities of miR403 across plants. (PPTX 239 KB)

Additional file 11: Figure S10: Ago3 targeting by miR403. (PPTX 51 KB)
