# Supplementary material for: Diversity, expression and mRNA targeting abilities of Argonaute-targeting miRNAs among selected vascular plants
Source: BMC Genomics. 2014 Dec 2;15(1):1049. doi: 10.1186/1471-2164-15-1049 (PMC4300679; doi:10.1186/1471-2164-15-1049)
Supplement: Supplementary file 1 — Additional file 1: Table S1: Plant species used for small RNA analysis. (PPTX 68 KB) [file 12864_2014_6764_MOESM1_ESM.pptx]

## Slide 1
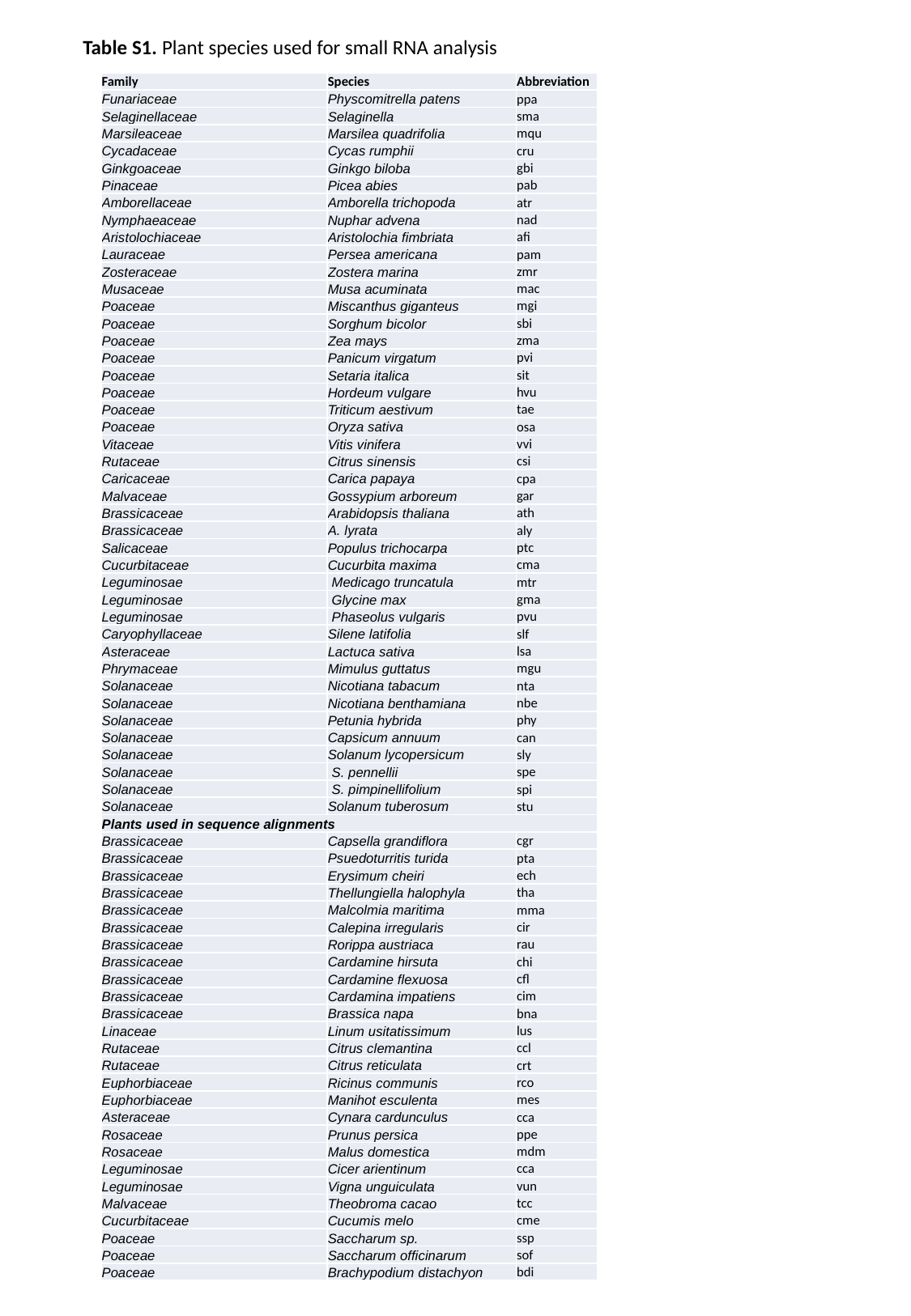

Table S1. Plant species used for small RNA analysis
| Family | Species | Abbreviation |
| --- | --- | --- |
| Funariaceae | Physcomitrella patens | ppa |
| Selaginellaceae | Selaginella | sma |
| Marsileaceae | Marsilea quadrifolia | mqu |
| Cycadaceae | Cycas rumphii | cru |
| Ginkgoaceae | Ginkgo biloba | gbi |
| Pinaceae | Picea abies | pab |
| Amborellaceae | Amborella trichopoda | atr |
| Nymphaeaceae | Nuphar advena | nad |
| Aristolochiaceae | Aristolochia fimbriata | afi |
| Lauraceae | Persea americana | pam |
| Zosteraceae | Zostera marina | zmr |
| Musaceae | Musa acuminata | mac |
| Poaceae | Miscanthus giganteus | mgi |
| Poaceae | Sorghum bicolor | sbi |
| Poaceae | Zea mays | zma |
| Poaceae | Panicum virgatum | pvi |
| Poaceae | Setaria italica | sit |
| Poaceae | Hordeum vulgare | hvu |
| Poaceae | Triticum aestivum | tae |
| Poaceae | Oryza sativa | osa |
| Vitaceae | Vitis vinifera | vvi |
| Rutaceae | Citrus sinensis | csi |
| Caricaceae | Carica papaya | cpa |
| Malvaceae | Gossypium arboreum | gar |
| Brassicaceae | Arabidopsis thaliana | ath |
| Brassicaceae | A. lyrata | aly |
| Salicaceae | Populus trichocarpa | ptc |
| Cucurbitaceae | Cucurbita maxima | cma |
| Leguminosae | Medicago truncatula | mtr |
| Leguminosae | Glycine max | gma |
| Leguminosae | Phaseolus vulgaris | pvu |
| Caryophyllaceae | Silene latifolia | slf |
| Asteraceae | Lactuca sativa | lsa |
| Phrymaceae | Mimulus guttatus | mgu |
| Solanaceae | Nicotiana tabacum | nta |
| Solanaceae | Nicotiana benthamiana | nbe |
| Solanaceae | Petunia hybrida | phy |
| Solanaceae | Capsicum annuum | can |
| Solanaceae | Solanum lycopersicum | sly |
| Solanaceae | S. pennellii | spe |
| Solanaceae | S. pimpinellifolium | spi |
| Solanaceae | Solanum tuberosum | stu |
| Plants used in sequence alignments | | |
| Brassicaceae | Capsella grandiflora | cgr |
| Brassicaceae | Psuedoturritis turida | pta |
| Brassicaceae | Erysimum cheiri | ech |
| Brassicaceae | Thellungiella halophyla | tha |
| Brassicaceae | Malcolmia maritima | mma |
| Brassicaceae | Calepina irregularis | cir |
| Brassicaceae | Rorippa austriaca | rau |
| Brassicaceae | Cardamine hirsuta | chi |
| Brassicaceae | Cardamine flexuosa | cfl |
| Brassicaceae | Cardamina impatiens | cim |
| Brassicaceae | Brassica napa | bna |
| Linaceae | Linum usitatissimum | lus |
| Rutaceae | Citrus clemantina | ccl |
| Rutaceae | Citrus reticulata | crt |
| Euphorbiaceae | Ricinus communis | rco |
| Euphorbiaceae | Manihot esculenta | mes |
| Asteraceae | Cynara cardunculus | cca |
| Rosaceae | Prunus persica | ppe |
| Rosaceae | Malus domestica | mdm |
| Leguminosae | Cicer arientinum | cca |
| Leguminosae | Vigna unguiculata | vun |
| Malvaceae | Theobroma cacao | tcc |
| Cucurbitaceae | Cucumis melo | cme |
| Poaceae | Saccharum sp. | ssp |
| Poaceae | Saccharum officinarum | sof |
| Poaceae | Brachypodium distachyon | bdi |
