# Supplementary material for: Diversity, expression and mRNA targeting abilities of Argonaute-targeting miRNAs among selected vascular plants
Source: BMC Genomics. 2014 Dec 2;15(1):1049. doi: 10.1186/1471-2164-15-1049 (PMC4300679; doi:10.1186/1471-2164-15-1049)
Supplement: Supplementary file 2 — Additional file 2: Figure S1: Phylogenetic analysis of precursors of miR168. (PPTX 62 KB) [file 12864_2014_6764_MOESM2_ESM.pptx]

## Slide 1
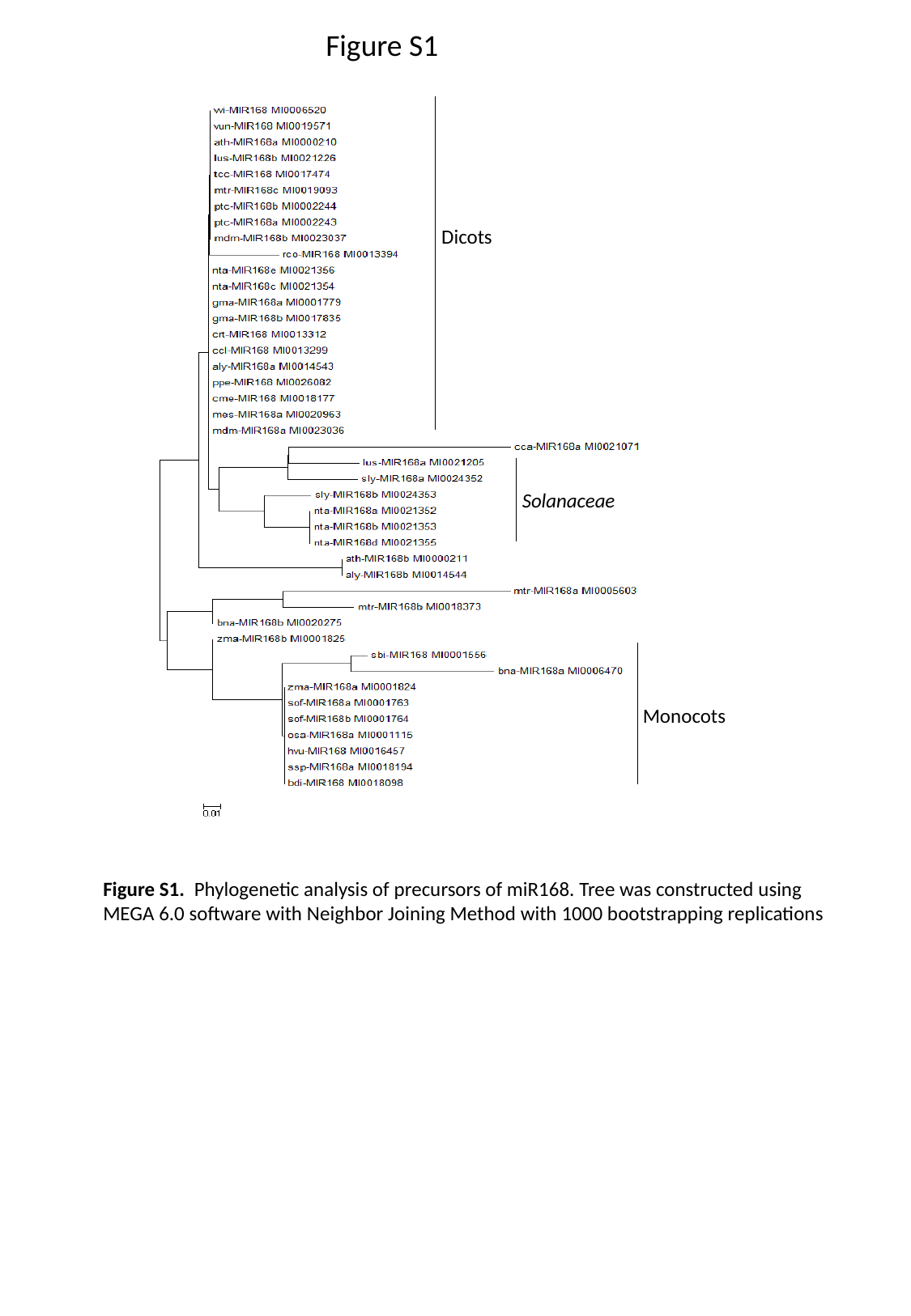

Figure S1
Dicots
Solanaceae
Monocots
Figure S1. Phylogenetic analysis of precursors of miR168. Tree was constructed using MEGA 6.0 software with Neighbor Joining Method with 1000 bootstrapping replications
