# Supplementary material for: Diversity, expression and mRNA targeting abilities of Argonaute-targeting miRNAs among selected vascular plants
Source: BMC Genomics. 2014 Dec 2;15(1):1049. doi: 10.1186/1471-2164-15-1049 (PMC4300679; doi:10.1186/1471-2164-15-1049)
Supplement: Supplementary file 3 — Additional file 3: Figure S2: Targeting abilities of miR168 among selected plants indicating mosaic targeting among multiple AGO1 members in monocots. (PPTX 675 KB) [file 12864_2014_6764_MOESM3_ESM.pptx]

## Slide 1
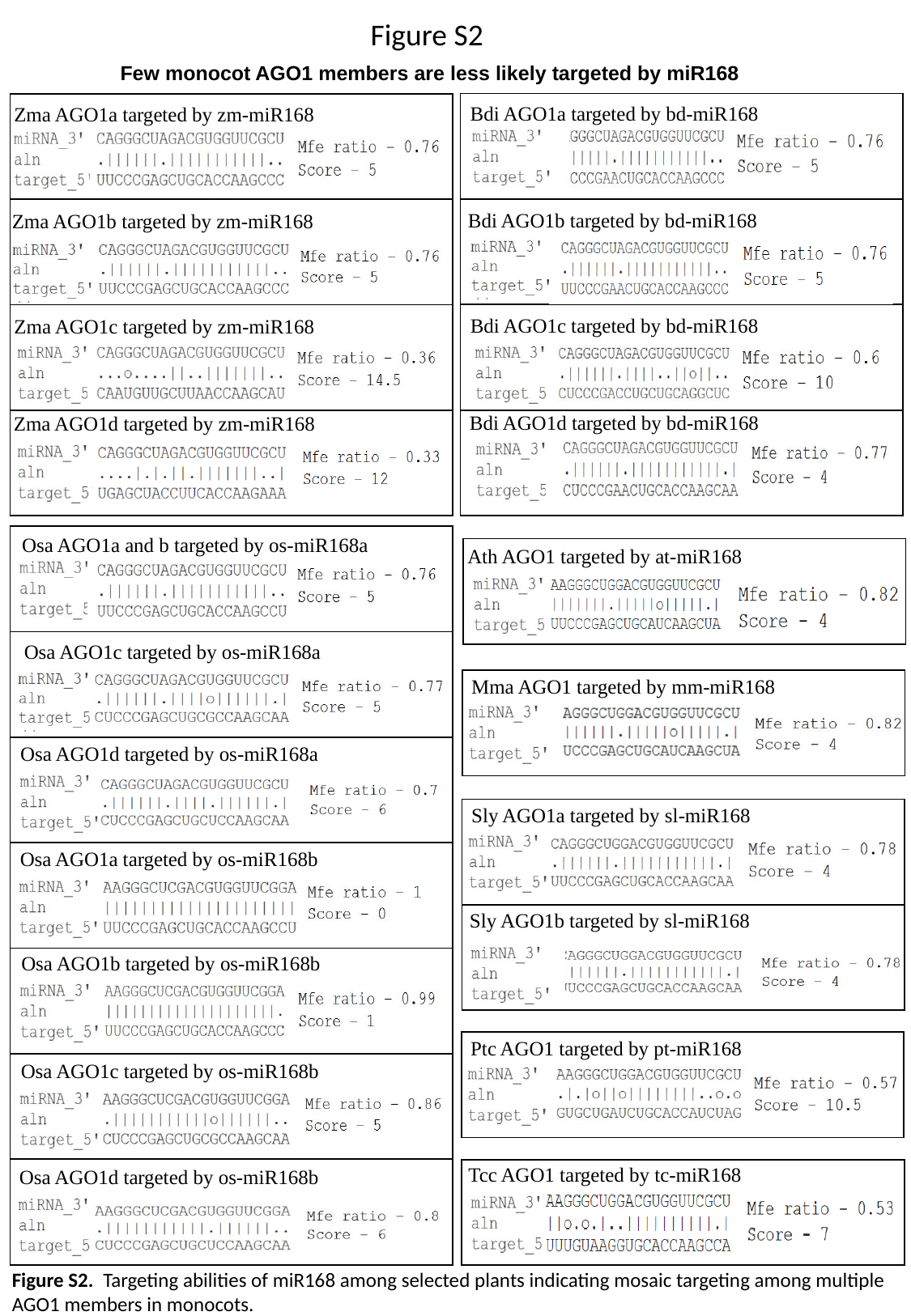

Figure S2
Few monocot AGO1 members are less likely targeted by miR168
| |
| --- |
| |
| |
| |
| |
| --- |
| |
| |
| |
Bdi AGO1a targeted by bd-miR168
Zma AGO1a targeted by zm-miR168
Bdi AGO1b targeted by bd-miR168
Zma AGO1b targeted by zm-miR168
Bdi AGO1c targeted by bd-miR168
Zma AGO1c targeted by zm-miR168
Bdi AGO1d targeted by bd-miR168
Zma AGO1d targeted by zm-miR168
| |
| --- |
| |
| |
| |
| |
| |
| |
Osa AGO1a and b targeted by os-miR168a
Ath AGO1 targeted by at-miR168
| |
| --- |
Osa AGO1c targeted by os-miR168a
Mma AGO1 targeted by mm-miR168
| |
| --- |
Osa AGO1d targeted by os-miR168a
Sly AGO1a targeted by sl-miR168
| |
| --- |
| |
Osa AGO1a targeted by os-miR168b
Sly AGO1b targeted by sl-miR168
Osa AGO1b targeted by os-miR168b
Ptc AGO1 targeted by pt-miR168
| |
| --- |
Osa AGO1c targeted by os-miR168b
Tcc AGO1 targeted by tc-miR168
Osa AGO1d targeted by os-miR168b
| |
| --- |
Figure S2. Targeting abilities of miR168 among selected plants indicating mosaic targeting among multiple AGO1 members in monocots.
