# Supplementary material for: Diversity, expression and mRNA targeting abilities of Argonaute-targeting miRNAs among selected vascular plants
Source: BMC Genomics. 2014 Dec 2;15(1):1049. doi: 10.1186/1471-2164-15-1049 (PMC4300679; doi:10.1186/1471-2164-15-1049)
Supplement: Supplementary file 4 — Additional file 4: Figure S3: Length of loops (distance between miRNA and miRNA*) among 58 miR168 precursors from diverse plants. (PPTX 94 KB) [file 12864_2014_6764_MOESM4_ESM.pptx]

## Slide 1
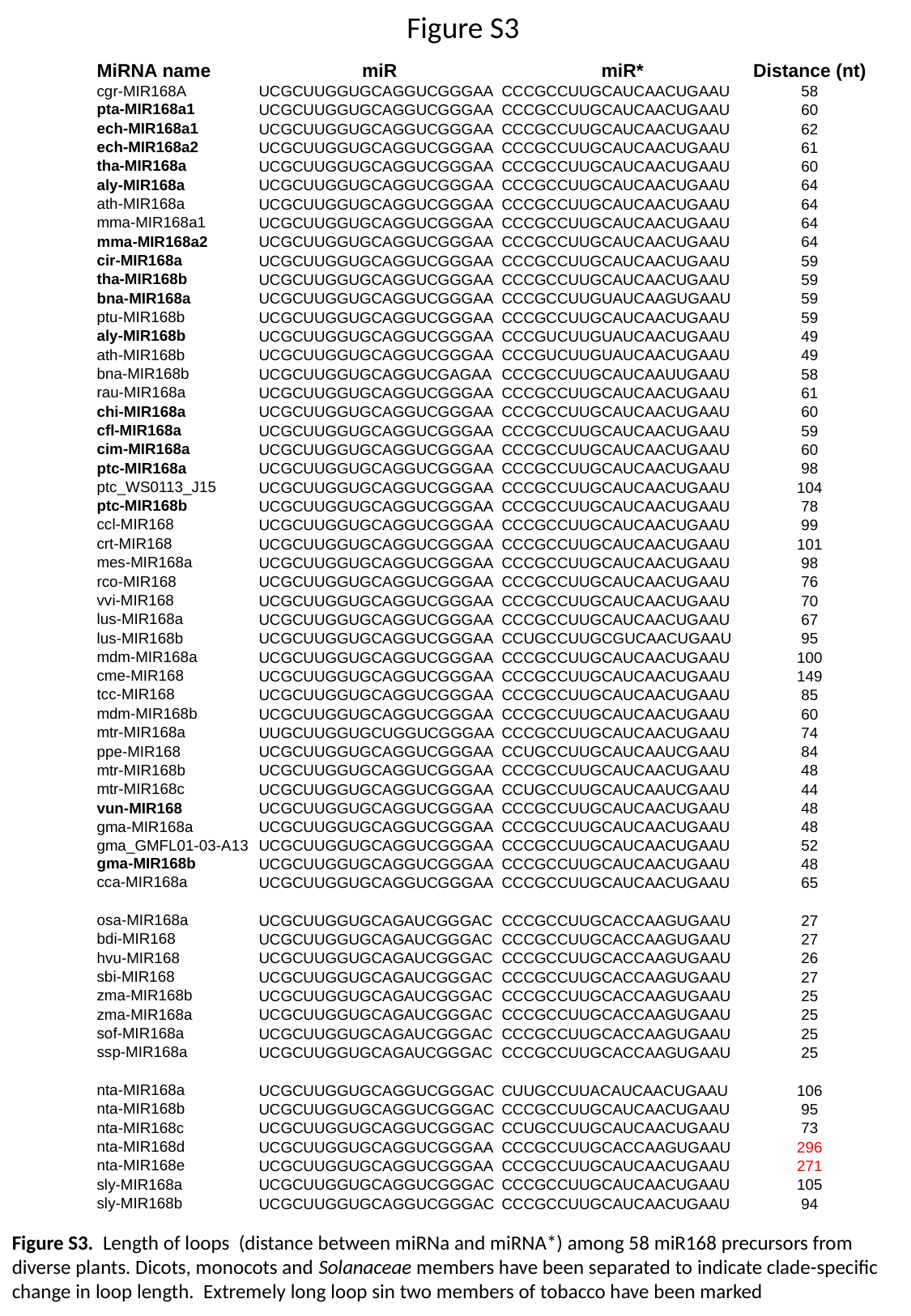

Figure S3
| MiRNA name | miR | miR\* | Distance (nt) |
| --- | --- | --- | --- |
| cgr-MIR168A | UCGCUUGGUGCAGGUCGGGAA | CCCGCCUUGCAUCAACUGAAU | 58 |
| pta-MIR168a1 | UCGCUUGGUGCAGGUCGGGAA | CCCGCCUUGCAUCAACUGAAU | 60 |
| ech-MIR168a1 | UCGCUUGGUGCAGGUCGGGAA | CCCGCCUUGCAUCAACUGAAU | 62 |
| ech-MIR168a2 | UCGCUUGGUGCAGGUCGGGAA | CCCGCCUUGCAUCAACUGAAU | 61 |
| tha-MIR168a | UCGCUUGGUGCAGGUCGGGAA | CCCGCCUUGCAUCAACUGAAU | 60 |
| aly-MIR168a | UCGCUUGGUGCAGGUCGGGAA | CCCGCCUUGCAUCAACUGAAU | 64 |
| ath-MIR168a | UCGCUUGGUGCAGGUCGGGAA | CCCGCCUUGCAUCAACUGAAU | 64 |
| mma-MIR168a1 | UCGCUUGGUGCAGGUCGGGAA | CCCGCCUUGCAUCAACUGAAU | 64 |
| mma-MIR168a2 | UCGCUUGGUGCAGGUCGGGAA | CCCGCCUUGCAUCAACUGAAU | 64 |
| cir-MIR168a | UCGCUUGGUGCAGGUCGGGAA | CCCGCCUUGCAUCAACUGAAU | 59 |
| tha-MIR168b | UCGCUUGGUGCAGGUCGGGAA | CCCGCCUUGCAUCAACUGAAU | 59 |
| bna-MIR168a | UCGCUUGGUGCAGGUCGGGAA | CCCGCCUUGUAUCAAGUGAAU | 59 |
| ptu-MIR168b | UCGCUUGGUGCAGGUCGGGAA | CCCGCCUUGCAUCAACUGAAU | 59 |
| aly-MIR168b | UCGCUUGGUGCAGGUCGGGAA | CCCGUCUUGUAUCAACUGAAU | 49 |
| ath-MIR168b | UCGCUUGGUGCAGGUCGGGAA | CCCGUCUUGUAUCAACUGAAU | 49 |
| bna-MIR168b | UCGCUUGGUGCAGGUCGAGAA | CCCGCCUUGCAUCAAUUGAAU | 58 |
| rau-MIR168a | UCGCUUGGUGCAGGUCGGGAA | CCCGCCUUGCAUCAACUGAAU | 61 |
| chi-MIR168a | UCGCUUGGUGCAGGUCGGGAA | CCCGCCUUGCAUCAACUGAAU | 60 |
| cfl-MIR168a | UCGCUUGGUGCAGGUCGGGAA | CCCGCCUUGCAUCAACUGAAU | 59 |
| cim-MIR168a | UCGCUUGGUGCAGGUCGGGAA | CCCGCCUUGCAUCAACUGAAU | 60 |
| ptc-MIR168a | UCGCUUGGUGCAGGUCGGGAA | CCCGCCUUGCAUCAACUGAAU | 98 |
| ptc\_WS0113\_J15 | UCGCUUGGUGCAGGUCGGGAA | CCCGCCUUGCAUCAACUGAAU | 104 |
| ptc-MIR168b | UCGCUUGGUGCAGGUCGGGAA | CCCGCCUUGCAUCAACUGAAU | 78 |
| ccl-MIR168 | UCGCUUGGUGCAGGUCGGGAA | CCCGCCUUGCAUCAACUGAAU | 99 |
| crt-MIR168 | UCGCUUGGUGCAGGUCGGGAA | CCCGCCUUGCAUCAACUGAAU | 101 |
| mes-MIR168a | UCGCUUGGUGCAGGUCGGGAA | CCCGCCUUGCAUCAACUGAAU | 98 |
| rco-MIR168 | UCGCUUGGUGCAGGUCGGGAA | CCCGCCUUGCAUCAACUGAAU | 76 |
| vvi-MIR168 | UCGCUUGGUGCAGGUCGGGAA | CCCGCCUUGCAUCAACUGAAU | 70 |
| lus-MIR168a | UCGCUUGGUGCAGGUCGGGAA | CCCGCCUUGCAUCAACUGAAU | 67 |
| lus-MIR168b | UCGCUUGGUGCAGGUCGGGAA | CCUGCCUUGCGUCAACUGAAU | 95 |
| mdm-MIR168a | UCGCUUGGUGCAGGUCGGGAA | CCCGCCUUGCAUCAACUGAAU | 100 |
| cme-MIR168 | UCGCUUGGUGCAGGUCGGGAA | CCCGCCUUGCAUCAACUGAAU | 149 |
| tcc-MIR168 | UCGCUUGGUGCAGGUCGGGAA | CCCGCCUUGCAUCAACUGAAU | 85 |
| mdm-MIR168b | UCGCUUGGUGCAGGUCGGGAA | CCCGCCUUGCAUCAACUGAAU | 60 |
| mtr-MIR168a | UUGCUUGGUGCUGGUCGGGAA | CCCGCCUUGCAUCAACUGAAU | 74 |
| ppe-MIR168 | UCGCUUGGUGCAGGUCGGGAA | CCUGCCUUGCAUCAAUCGAAU | 84 |
| mtr-MIR168b | UCGCUUGGUGCAGGUCGGGAA | CCCGCCUUGCAUCAACUGAAU | 48 |
| mtr-MIR168c | UCGCUUGGUGCAGGUCGGGAA | CCUGCCUUGCAUCAAUCGAAU | 44 |
| vun-MIR168 | UCGCUUGGUGCAGGUCGGGAA | CCCGCCUUGCAUCAACUGAAU | 48 |
| gma-MIR168a | UCGCUUGGUGCAGGUCGGGAA | CCCGCCUUGCAUCAACUGAAU | 48 |
| gma\_GMFL01-03-A13 | UCGCUUGGUGCAGGUCGGGAA | CCCGCCUUGCAUCAACUGAAU | 52 |
| gma-MIR168b | UCGCUUGGUGCAGGUCGGGAA | CCCGCCUUGCAUCAACUGAAU | 48 |
| cca-MIR168a | UCGCUUGGUGCAGGUCGGGAA | CCCGCCUUGCAUCAACUGAAU | 65 |
| | | | |
| osa-MIR168a | UCGCUUGGUGCAGAUCGGGAC | CCCGCCUUGCACCAAGUGAAU | 27 |
| bdi-MIR168 | UCGCUUGGUGCAGAUCGGGAC | CCCGCCUUGCACCAAGUGAAU | 27 |
| hvu-MIR168 | UCGCUUGGUGCAGAUCGGGAC | CCCGCCUUGCACCAAGUGAAU | 26 |
| sbi-MIR168 | UCGCUUGGUGCAGAUCGGGAC | CCCGCCUUGCACCAAGUGAAU | 27 |
| zma-MIR168b | UCGCUUGGUGCAGAUCGGGAC | CCCGCCUUGCACCAAGUGAAU | 25 |
| zma-MIR168a | UCGCUUGGUGCAGAUCGGGAC | CCCGCCUUGCACCAAGUGAAU | 25 |
| sof-MIR168a | UCGCUUGGUGCAGAUCGGGAC | CCCGCCUUGCACCAAGUGAAU | 25 |
| ssp-MIR168a | UCGCUUGGUGCAGAUCGGGAC | CCCGCCUUGCACCAAGUGAAU | 25 |
| | | | |
| nta-MIR168a | UCGCUUGGUGCAGGUCGGGAC | CUUGCCUUACAUCAACUGAAU | 106 |
| nta-MIR168b | UCGCUUGGUGCAGGUCGGGAC | CCCGCCUUGCAUCAACUGAAU | 95 |
| nta-MIR168c | UCGCUUGGUGCAGGUCGGGAC | CCUGCCUUGCAUCAACUGAAU | 73 |
| nta-MIR168d | UCGCUUGGUGCAGGUCGGGAA | CCCGCCUUGCACCAAGUGAAU | 296 |
| nta-MIR168e | UCGCUUGGUGCAGGUCGGGAA | CCCGCCUUGCAUCAACUGAAU | 271 |
| sly-MIR168a | UCGCUUGGUGCAGGUCGGGAC | CCCGCCUUGCAUCAACUGAAU | 105 |
| sly-MIR168b | UCGCUUGGUGCAGGUCGGGAC | CCCGCCUUGCAUCAACUGAAU | 94 |
Figure S3. Length of loops (distance between miRNa and miRNA*) among 58 miR168 precursors from diverse plants. Dicots, monocots and Solanaceae members have been separated to indicate clade-specific change in loop length. Extremely long loop sin two members of tobacco have been marked
