# Supplementary material for: Diversity, expression and mRNA targeting abilities of Argonaute-targeting miRNAs among selected vascular plants
Source: BMC Genomics. 2014 Dec 2;15(1):1049. doi: 10.1186/1471-2164-15-1049 (PMC4300679; doi:10.1186/1471-2164-15-1049)
Supplement: Supplementary file 5 — Additional file 5: Figure S4: Secondary structures of 58 precursors of miR168 indicating clade-specific changes in the loop region. (PPTX 620 KB) [file 12864_2014_6764_MOESM5_ESM.pptx]

## Slide 1
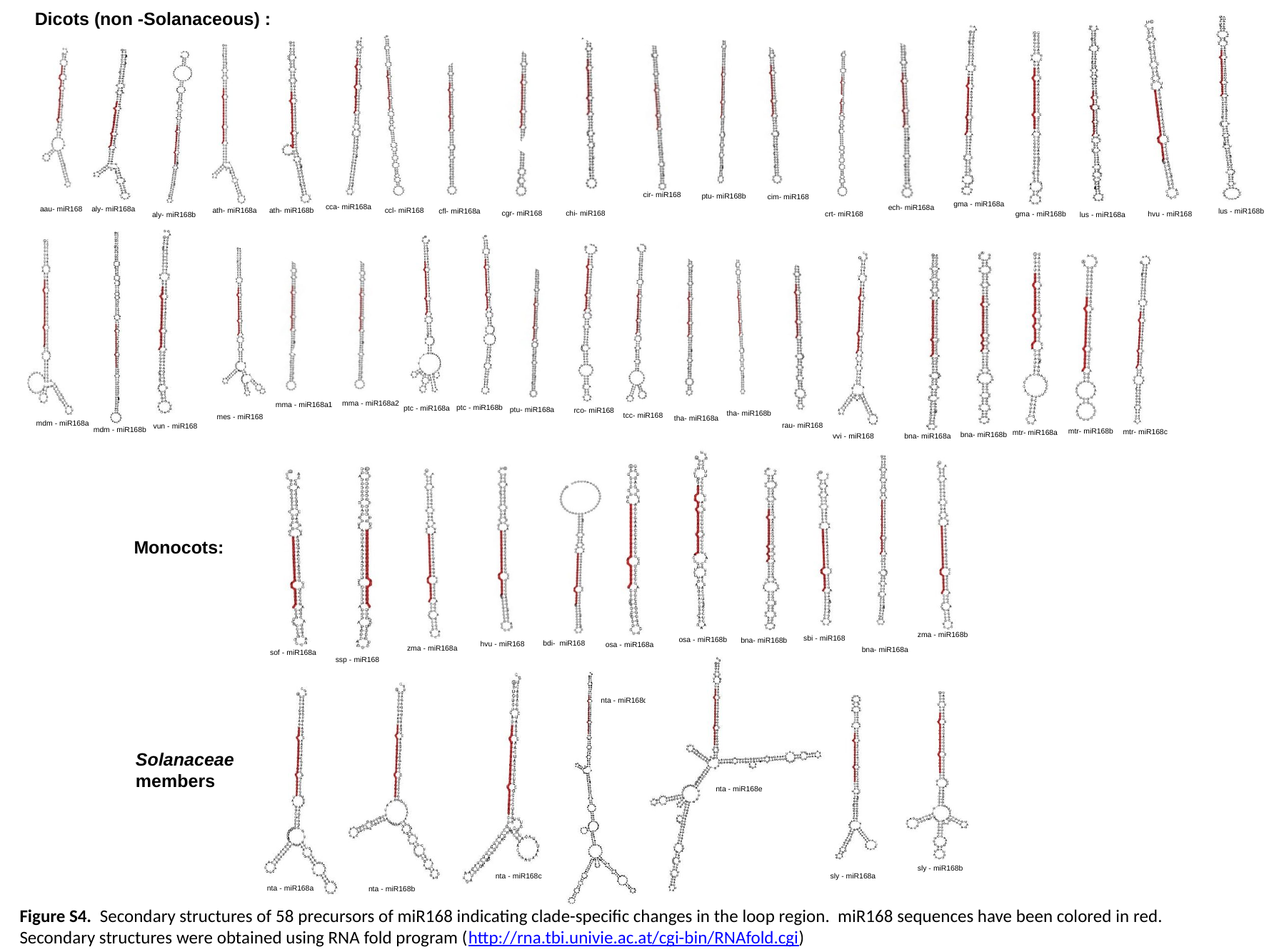

Dicots (non -Solanaceous) :
cir- miR168
ptu- miR168b
cim- miR168
gma - miR168a
cca- miR168a
ech- miR168a
aly- miR168a
aau- miR168
ath- miR168a
ccl- miR168
ath- miR168b
cfl- miR168a
lus - miR168b
chi- miR168
cgr- miR168
hvu - miR168
gma - miR168b
crt- miR168
aly- miR168b
lus - miR168a
mma - miR168a2
mma - miR168a1
ptc - miR168b
ptc - miR168a
ptu- miR168a
rco- miR168
tha- miR168b
tcc- miR168
mes - miR168
tha- miR168a
mdm - miR168a
rau- miR168
vun - miR168
mdm - miR168b
mtr- miR168b
mtr- miR168c
mtr- miR168a
bna- miR168b
vvi - miR168
bna- miR168a
Monocots:
zma - miR168b
sbi - miR168
osa - miR168b
bna- miR168b
bdi- miR168
hvu - miR168
osa - miR168a
zma - miR168a
bna- miR168a
sof - miR168a
ssp - miR168
nta - miR168d
Solanaceae members
nta - miR168e
sly - miR168b
sly - miR168a
nta - miR168c
nta - miR168a
nta - miR168b
Figure S4. Secondary structures of 58 precursors of miR168 indicating clade-specific changes in the loop region. miR168 sequences have been colored in red. Secondary structures were obtained using RNA fold program (http://rna.tbi.univie.ac.at/cgi-bin/RNAfold.cgi)
