# Supplementary material for: Diversity, expression and mRNA targeting abilities of Argonaute-targeting miRNAs among selected vascular plants
Source: BMC Genomics. 2014 Dec 2;15(1):1049. doi: 10.1186/1471-2164-15-1049 (PMC4300679; doi:10.1186/1471-2164-15-1049)
Supplement: Supplementary file 6 — Additional file 6: Figure S5: Multiple sequence alignment of miR168 precursors from Solanaceae indicating site of MITE insertion. (PPTX 79 KB) [file 12864_2014_6764_MOESM6_ESM.pptx]

## Slide 1
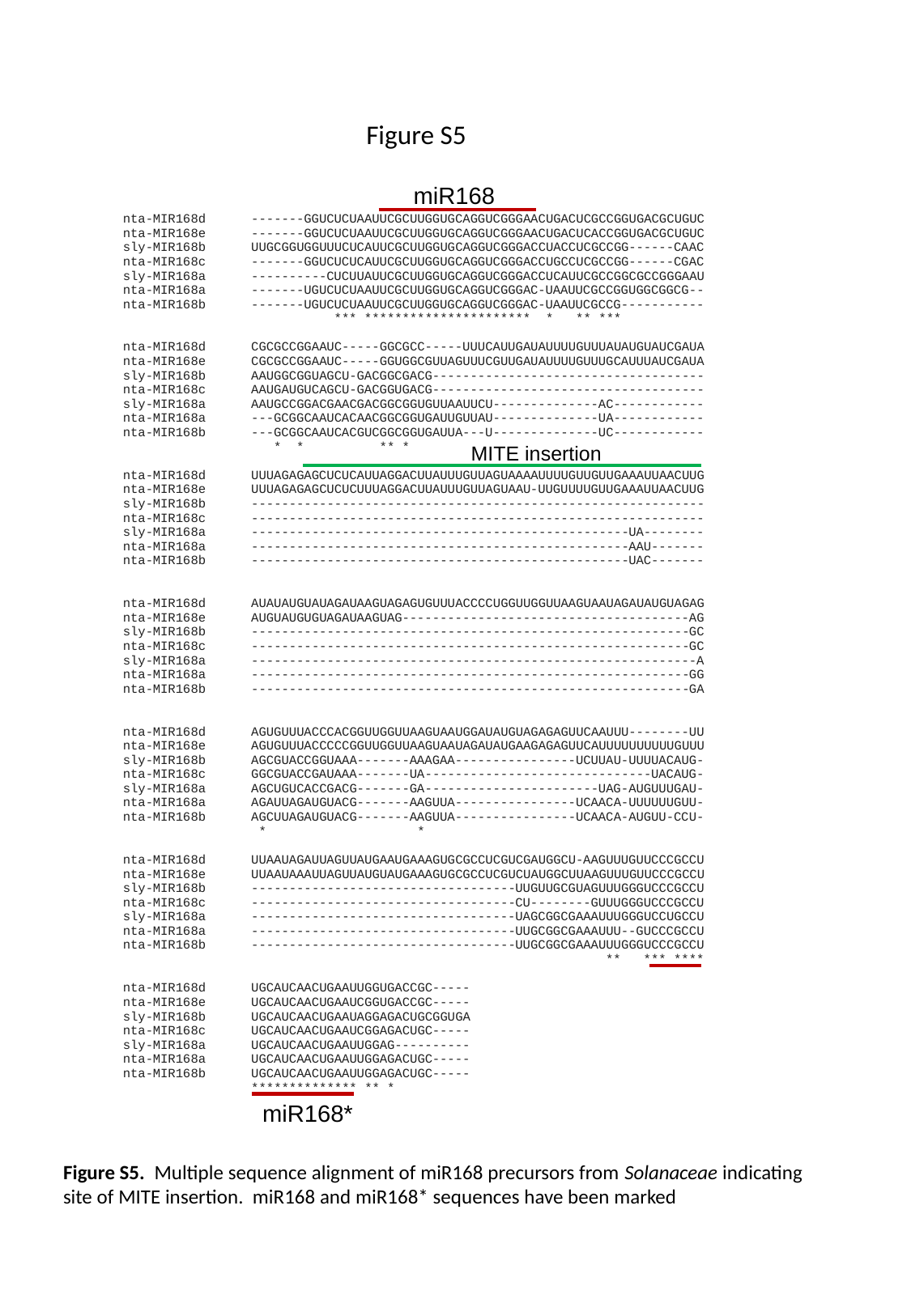

Figure S5
miR168
MITE insertion
miR168*
Figure S5. Multiple sequence alignment of miR168 precursors from Solanaceae indicating site of MITE insertion. miR168 and miR168* sequences have been marked
