# Supplementary material for: Diversity, expression and mRNA targeting abilities of Argonaute-targeting miRNAs among selected vascular plants
Source: BMC Genomics. 2014 Dec 2;15(1):1049. doi: 10.1186/1471-2164-15-1049 (PMC4300679; doi:10.1186/1471-2164-15-1049)
Supplement: Supplementary file 7 — Additional file 7: Figure S6: Abundance and sequence diversity of miR168 members across plant families in reproductive tissues. (PPTX 88 KB) [file 12864_2014_6764_MOESM7_ESM.pptx]

## Slide 1
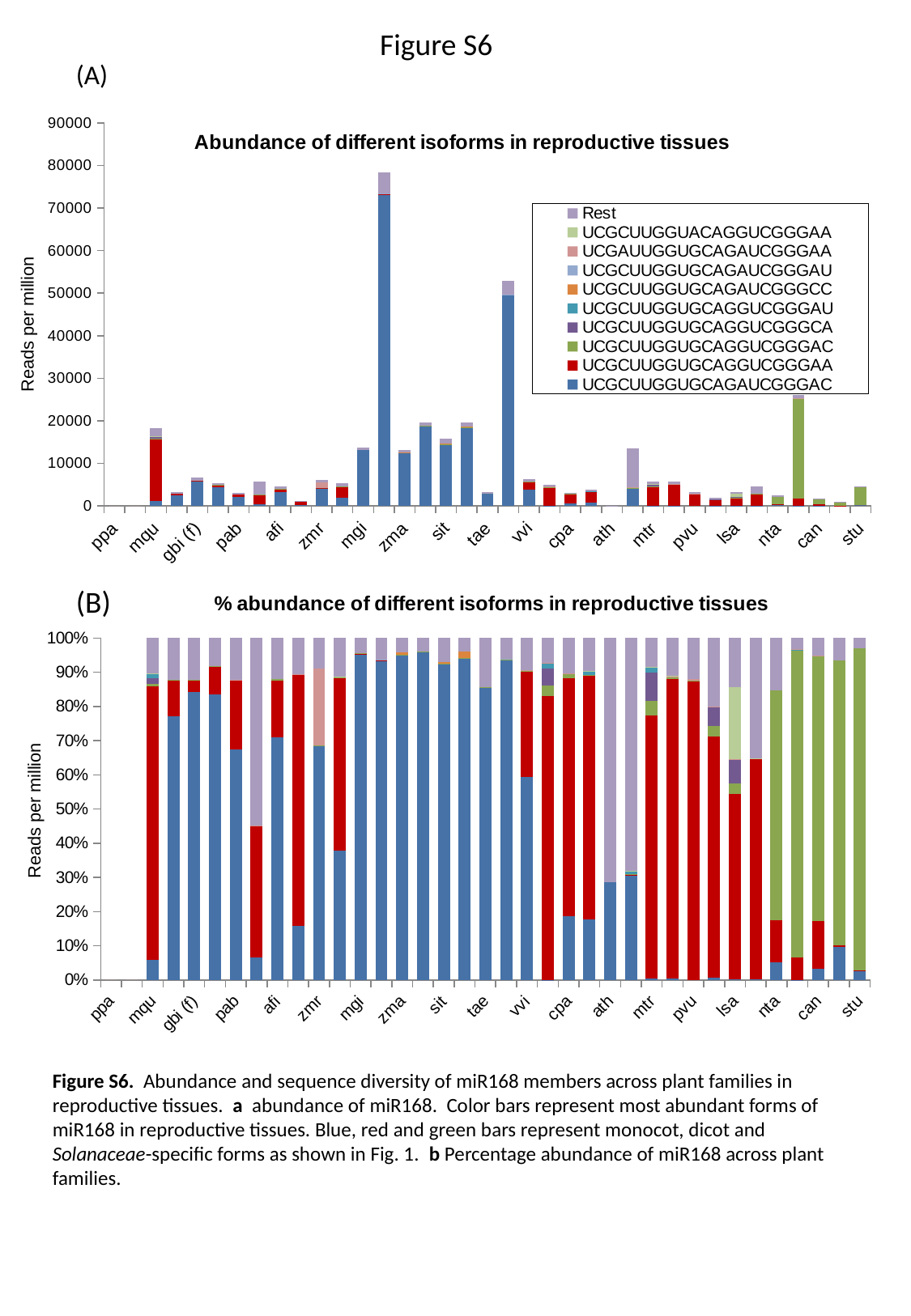

Figure S6
(A)
### Chart
| Category | UCGCUUGGUGCAGAUCGGGAC | UCGCUUGGUGCAGGUCGGGAA | UCGCUUGGUGCAGGUCGGGAC | UCGCUUGGUGCAGGUCGGGCA | UCGCUUGGUGCAGGUCGGGAU | UCGCUUGGUGCAGAUCGGGCC | UCGCUUGGUGCAGAUCGGGAU | UCGAUUGGUGCAGAUCGGGAA | UCGCUUGGUACAGGUCGGGAA | Rest |
|---|---|---|---|---|---|---|---|---|---|---|
| ppa | 0.0 | 0.0 | 0.0 | 0.0 | 0.0 | 0.0 | 0.0 | 0.0 | 0.0 | 0.0 |
| sma | 0.0 | 0.0 | 0.0 | 0.0 | 0.0 | 0.0 | 0.0 | 0.0 | 0.0 | 0.0 |
| mqu | 1052.7901316780428 | 14572.581476284477 | 141.1119303002196 | 325.0330978825283 | 247.34225985207033 | None | None | 4.756581920232121 | 6.342109226976162 | 1861.4090581175035 |
| cru | 2480.355032248586 | 341.47897455716924 | 2.6471238337765057 | None | None | None | None | None | None | 395.74501314958763 |
| gbi (f) | 5625.952053212951 | 239.85923484904086 | 3.5799885798364306 | None | None | None | None | None | None | 814.447401912788 |
| gbi (m) | 4465.195485975896 | 431.7902548571359 | 4.035422942590055 | None | None | None | None | None | None | 437.843389271021 |
| pab | 2123.2379096525665 | 636.4081798162865 | 1.877310264944798 | None | None | None | None | 1.877310264944798 | None | 381.093983783794 |
| nad | 378.5418844440123 | 2191.375496185062 | 6.9457226503488485 | None | None | None | None | 6.9457226503488485 | None | 3129.0480539821565 |
| afi | 3265.216412793205 | 757.2170842023893 | 24.83912900465094 | None | None | None | None | None | None | 546.4608381023206 |
| pam | 193.64327862004143 | 904.7565171479463 | 1.8131393129217361 | None | None | None | None | 0.7252557251686944 | None | 129.0955190800276 |
| zmr | 4105.819083980806 | 11.141978518265416 | 3.7139928394218056 | None | None | None | None | 1357.46438280867 | None | 531.1009760373182 |
| mac | 1992.792577692661 | 2640.7558084761645 | 25.88976282819769 | None | None | None | None | 1.4383201571220938 | 0.7191600785610469 | 589.7112644200585 |
| mgi | 13147.155480552028 | 1.800404730983525 | 6.6014840136062585 | None | None | None | None | None | None | 617.538822727349 |
| sbi | 73265.78546779537 | 69.56833194426268 | 23.419042436682485 | None | None | None | 139.13666388852536 | None | None | 4870.472031464172 |
| zma | 12404.850621273261 | None | 4.724184906315118 | None | None | 88.04162779950902 | None | None | None | 543.7107355813581 |
| pvi | 18913.953222537268 | None | 6.691257036274977 | None | None | None | None | None | None | 751.6512070748892 |
| sit | 14523.392516779932 | None | 4.85894697784541 | None | None | 60.51597599680193 | None | None | None | 1115.7909150943187 |
| hvu | 18342.216581179782 | None | 20.21296096877237 | None | None | 368.015289362476 | None | None | None | 782.0324898952621 |
| tae | 2780.6629808520843 | None | 2.6615582492003678 | None | None | None | None | None | None | 465.1073040477643 |
| osa | 49523.246677052564 | None | 9.758736704209177 | None | None | None | 49.57438245738262 | None | None | 3240.290935265615 |
| vvi | 3710.6481402279423 | 1931.4530891274076 | 23.950702610968737 | None | None | None | None | 5.132293416636158 | None | 583.37068502431 |
| csi | 3.53613291333504 | 4129.496016192659 | 150.6392621080727 | 252.47989001212184 | 70.7226582667008 | None | None | 11.315625322672128 | None | 361.39278374284106 |
| cpa | 572.3069897085907 | 2153.859049652449 | 38.53919122616772 | None | None | None | None | 1.9269595613083863 | None | 317.9483276158837 |
| gar | 692.155117641405 | 2773.6134741193905 | 2.4965017768851396 | None | 40.880216596494165 | None | None | 3.74475266532771 | 1.2482508884425698 | 371.0425765895539 |
| ath | 10.691356572778737 | None | None | None | None | None | None | None | None | 26.72839143194684 |
| cma | 4104.950917387425 | 25.25691899264182 | 23.85375682638394 | None | 131.19566254511167 | None | None | 11.315625322672128 | 2.1047432493868183 | 9154.2299726664 |
| mtr | 23.00425857623158 | 4347.1077721630345 | 239.1048694438616 | 457.9938752904288 | 97.59382426280065 | None | None | 2.091296234202871 | 1.3941974894685807 | 472.63294892984885 |
| gma | 19.59017356893782 | 5039.027979121229 | 18.50183059288572 | None | None | None | None | 30.473603329458832 | 3.2650289281563034 | 629.0622401581145 |
| pvu | None | 2843.267934885715 | 11.087266643835111 | None | None | None | None | 12.31918515981679 | None | 389.2862510502106 |
| sla | 13.473948516835302 | 1377.5130895446914 | 61.02906092919519 | 110.96192896217308 | None | None | None | 1.5851704137453295 | None | 389.1593365744784 |
| lsa | 3.728908362077002 | 1789.876013796961 | 109.07056959075231 | 233.056772629813 | None | None | None | 0.9322270905192505 | 691.7125011652839 | 474.50358907429853 |
| mgu | 11.667131661044463 | 2914.246582291323 | 8.116265503335278 | None | None | None | None | None | 1.5217997818753646 | 1597.3825043751744 |
| nta | 124.41145872435355 | 292.4384288405782 | 1605.5513250892868 | None | None | None | None | None | None | 362.15174622922456 |
| phy | 8.75470112356023 | 1702.3365391640045 | 23456.259399982973 | None | 87.24512498996228 | None | None | 9.056587369200237 | 0.3018862456400079 | 848.6022364940623 |
| can | 58.48556349897116 | 241.1743398048972 | 1357.7455010137498 | None | None | None | None | 0.3144385134353288 | None | 88.67166078876272 |
| sly | 86.93472998752802 | 3.3996821782832183 | 749.8727547527557 | None | None | None | None | None | None | 59.737272561262266 |
| stu | 119.11293190061289 | 1.7711960133920133 | 4232.272874000216 | None | None | None | None | None | None | 128.85450997426895 |Reads per million
### Chart
| Category | UCGCUUGGUGCAGAUCGGGAC | UCGCUUGGUGCAGGUCGGGAA | UCGCUUGGUGCAGGUCGGGAC | UCGCUUGGUGCAGGUCGGGCA | UCGCUUGGUGCAGGUCGGGAU | UCGCUUGGUGCAGAUCGGGCC | UCGCUUGGUGCAGAUCGGGAU | UCGAUUGGUGCAGAUCGGGAA | UCGCUUGGUACAGGUCGGGAA | Rest |
|---|---|---|---|---|---|---|---|---|---|---|
| ppa | 0.0 | 0.0 | 0.0 | 0.0 | 0.0 | 0.0 | 0.0 | 0.0 | 0.0 | 0.0 |
| sma | 0.0 | 0.0 | 0.0 | 0.0 | 0.0 | 0.0 | 0.0 | 0.0 | 0.0 | 0.0 |
| mqu | 1052.7901316780428 | 14572.581476284477 | 141.1119303002196 | 325.0330978825283 | 247.34225985207033 | None | None | 4.756581920232121 | 6.342109226976162 | 1861.4090581175035 |
| cru | 2480.355032248586 | 341.47897455716924 | 2.6471238337765057 | None | None | None | None | None | None | 395.74501314958763 |
| gbi (f) | 5625.952053212951 | 239.85923484904086 | 3.5799885798364306 | None | None | None | None | None | None | 814.447401912788 |
| gbi (m) | 4465.195485975896 | 431.7902548571359 | 4.035422942590055 | None | None | None | None | None | None | 437.843389271021 |
| pab | 2123.2379096525665 | 636.4081798162865 | 1.877310264944798 | None | None | None | None | 1.877310264944798 | None | 381.093983783794 |
| nad | 378.5418844440123 | 2191.375496185062 | 6.9457226503488485 | None | None | None | None | 6.9457226503488485 | None | 3129.0480539821565 |
| afi | 3265.216412793205 | 757.2170842023893 | 24.83912900465094 | None | None | None | None | None | None | 546.4608381023206 |
| pam | 193.64327862004143 | 904.7565171479463 | 1.8131393129217361 | None | None | None | None | 0.7252557251686944 | None | 129.0955190800276 |
| zmr | 4105.819083980806 | 11.141978518265416 | 3.7139928394218056 | None | None | None | None | 1357.46438280867 | None | 531.1009760373182 |
| mac | 1992.792577692661 | 2640.7558084761645 | 25.88976282819769 | None | None | None | None | 1.4383201571220938 | 0.7191600785610469 | 589.7112644200585 |
| mgi | 13147.155480552028 | 1.800404730983525 | 6.6014840136062585 | None | None | None | None | None | None | 617.538822727349 |
| sbi | 73265.78546779537 | 69.56833194426268 | 23.419042436682485 | None | None | None | 139.13666388852536 | None | None | 4870.472031464172 |
| zma | 12404.850621273261 | None | 4.724184906315118 | None | None | 88.04162779950902 | None | None | None | 543.7107355813581 |
| pvi | 18913.953222537268 | None | 6.691257036274977 | None | None | None | None | None | None | 751.6512070748892 |
| sit | 14523.392516779932 | None | 4.85894697784541 | None | None | 60.51597599680193 | None | None | None | 1115.7909150943187 |
| hvu | 18342.216581179782 | None | 20.21296096877237 | None | None | 368.015289362476 | None | None | None | 782.0324898952621 |
| tae | 2780.6629808520843 | None | 2.6615582492003678 | None | None | None | None | None | None | 465.1073040477643 |
| osa | 49523.246677052564 | None | 9.758736704209177 | None | None | None | 49.57438245738262 | None | None | 3240.290935265615 |
| vvi | 3710.6481402279423 | 1931.4530891274076 | 23.950702610968737 | None | None | None | None | 5.132293416636158 | None | 583.37068502431 |
| csi | 3.53613291333504 | 4129.496016192659 | 150.6392621080727 | 252.47989001212184 | 70.7226582667008 | None | None | 11.315625322672128 | None | 361.39278374284106 |
| cpa | 572.3069897085907 | 2153.859049652449 | 38.53919122616772 | None | None | None | None | 1.9269595613083863 | None | 317.9483276158837 |
| gar | 692.155117641405 | 2773.6134741193905 | 2.4965017768851396 | None | 40.880216596494165 | None | None | 3.74475266532771 | 1.2482508884425698 | 371.0425765895539 |
| ath | 10.691356572778737 | None | None | None | None | None | None | None | None | 26.72839143194684 |
| cma | 4104.950917387425 | 25.25691899264182 | 23.85375682638394 | None | 131.19566254511167 | None | None | 11.315625322672128 | 2.1047432493868183 | 9154.2299726664 |
| mtr | 23.00425857623158 | 4347.1077721630345 | 239.1048694438616 | 457.9938752904288 | 97.59382426280065 | None | None | 2.091296234202871 | 1.3941974894685807 | 472.63294892984885 |
| gma | 19.59017356893782 | 5039.027979121229 | 18.50183059288572 | None | None | None | None | 30.473603329458832 | 3.2650289281563034 | 629.0622401581145 |
| pvu | None | 2843.267934885715 | 11.087266643835111 | None | None | None | None | 12.31918515981679 | None | 389.2862510502106 |
| sla | 13.473948516835302 | 1377.5130895446914 | 61.02906092919519 | 110.96192896217308 | None | None | None | 1.5851704137453295 | None | 389.1593365744784 |
| lsa | 3.728908362077002 | 1789.876013796961 | 109.07056959075231 | 233.056772629813 | None | None | None | 0.9322270905192505 | 691.7125011652839 | 474.50358907429853 |
| mgu | 11.667131661044463 | 2914.246582291323 | 8.116265503335278 | None | None | None | None | None | 1.5217997818753646 | 1597.3825043751744 |
| nta | 124.41145872435355 | 292.4384288405782 | 1605.5513250892868 | None | None | None | None | None | None | 362.15174622922456 |
| phy | 8.75470112356023 | 1702.3365391640045 | 23456.259399982973 | None | 87.24512498996228 | None | None | 9.056587369200237 | 0.3018862456400079 | 848.6022364940623 |
| can | 58.48556349897116 | 241.1743398048972 | 1357.7455010137498 | None | None | None | None | 0.3144385134353288 | None | 88.67166078876272 |
| sly | 86.93472998752802 | 3.3996821782832183 | 749.8727547527557 | None | None | None | None | None | None | 59.737272561262266 |
| stu | 119.11293190061289 | 1.7711960133920133 | 4232.272874000216 | None | None | None | None | None | None | 128.85450997426895 |(B)
Reads per million
Figure S6. Abundance and sequence diversity of miR168 members across plant families in reproductive tissues. a abundance of miR168. Color bars represent most abundant forms of miR168 in reproductive tissues. Blue, red and green bars represent monocot, dicot and Solanaceae-specific forms as shown in Fig. 1. b Percentage abundance of miR168 across plant families.
