# Supplementary material for: Diversity, expression and mRNA targeting abilities of Argonaute-targeting miRNAs among selected vascular plants
Source: BMC Genomics. 2014 Dec 2;15(1):1049. doi: 10.1186/1471-2164-15-1049 (PMC4300679; doi:10.1186/1471-2164-15-1049)
Supplement: Supplementary file 8 — Additional file 8: Figure S7: miR168 is not significantly induced in rice upon infection with viruses. (PPTX 63 KB) [file 12864_2014_6764_MOESM8_ESM.pptx]

## Slide 1
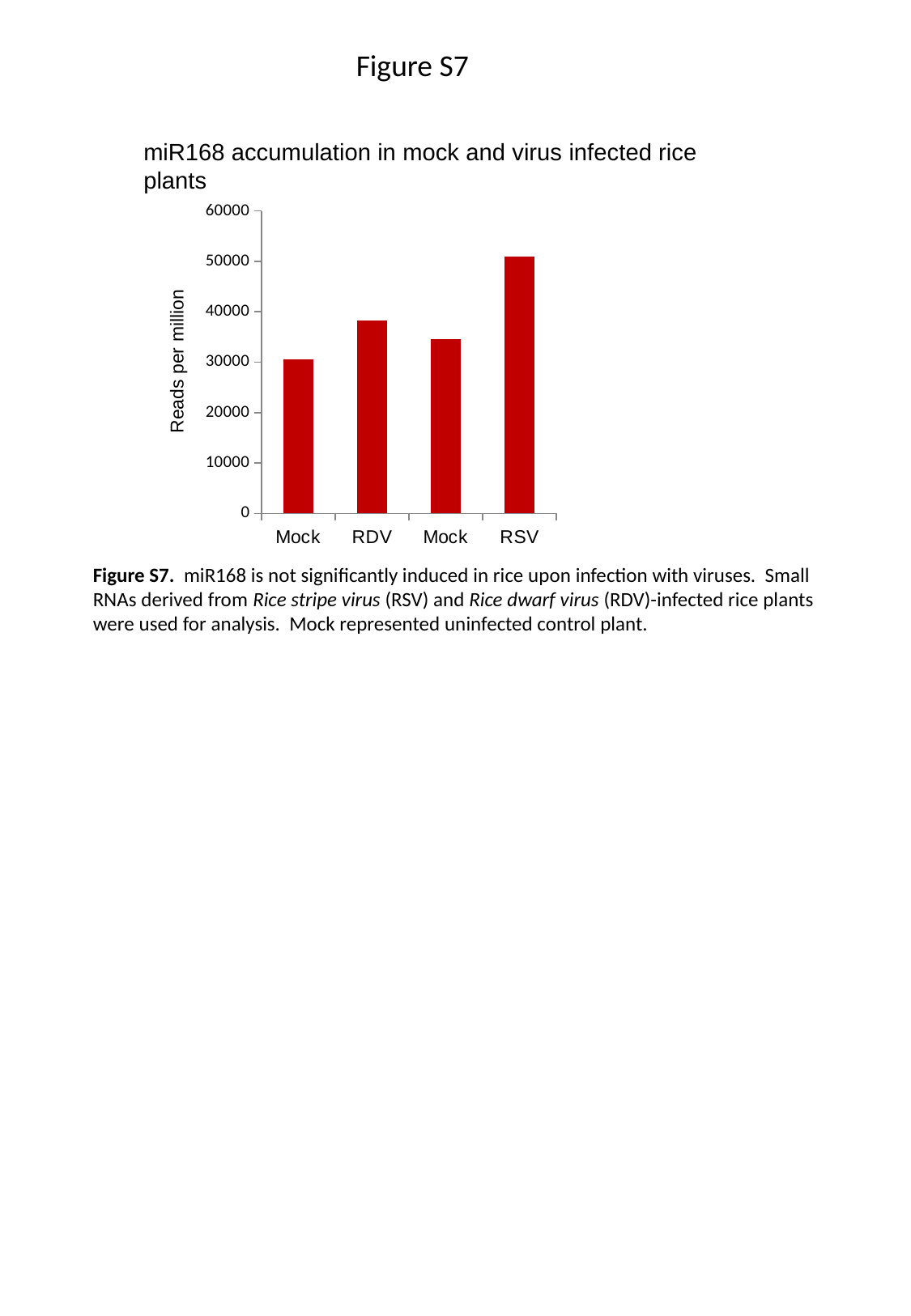

Figure S7
miR168 accumulation in mock and virus infected rice plants
### Chart
| Category | |
|---|---|
| Mock | 30643.333333333332 |
| RDV | 38322.6666666667 |
| Mock | 34504.666666666664 |
| RSV | 50927.6666666667 |Reads per million
Figure S7. miR168 is not significantly induced in rice upon infection with viruses. Small RNAs derived from Rice stripe virus (RSV) and Rice dwarf virus (RDV)-infected rice plants were used for analysis. Mock represented uninfected control plant.
