# Supplementary material for: Diversity, expression and mRNA targeting abilities of Argonaute-targeting miRNAs among selected vascular plants
Source: BMC Genomics. 2014 Dec 2;15(1):1049. doi: 10.1186/1471-2164-15-1049 (PMC4300679; doi:10.1186/1471-2164-15-1049)
Supplement: Supplementary file 9 — Additional file 9: Figure S8: Abundance and sequence diversity of miR403 members across plant families in reproductive tissues. (PPTX 6 MB) [file 12864_2014_6764_MOESM9_ESM.pptx]

## Slide 1
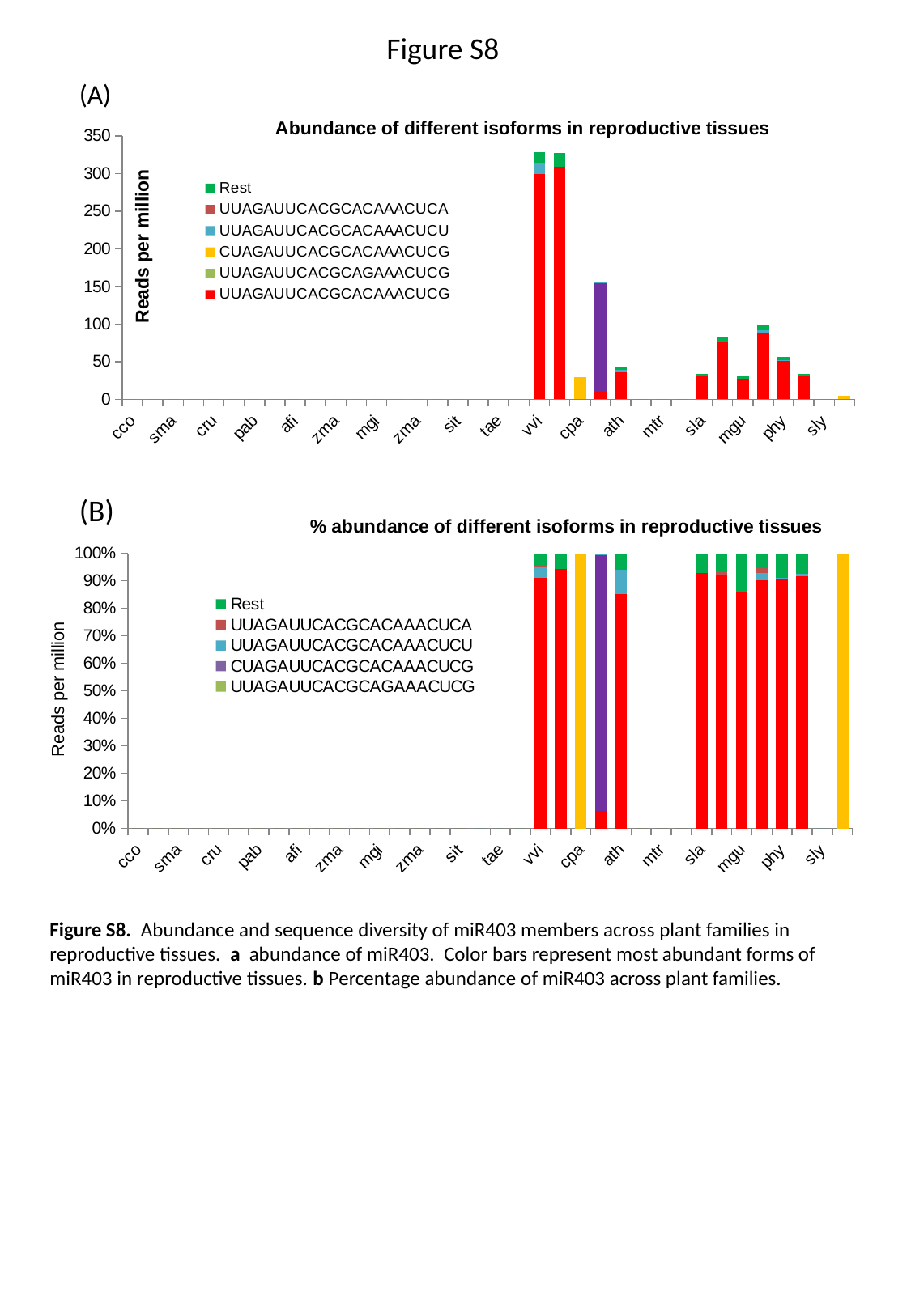

Figure S8
(A)
### Chart
| Category | UUAGAUUCACGCACAAACUCG | UUAGAUUCACGCAGAAACUCG | CUAGAUUCACGCACAAACUCG | UUAGAUUCACGCACAAACUCU | UUAGAUUCACGCACAAACUCA | Rest |
|---|---|---|---|---|---|---|
| cco | 0.0 | 0.0 | 0.0 | 0.0 | 0.0 | 0.0 |
| ppa | 0.0 | 0.0 | 0.0 | 0.0 | 0.0 | 0.0 |
| sma | 0.0 | 0.0 | 0.0 | 0.0 | 0.0 | 0.0 |
| mqu | 0.0 | 0.0 | 0.0 | 0.0 | 0.0 | 0.0 |
| cru | 0.0 | 0.0 | 0.0 | 0.0 | 0.0 | 0.0 |
| gbi | 0.0 | 0.0 | 0.0 | 0.0 | 0.0 | 0.0 |
| pab | 0.0 | 0.0 | 0.0 | 0.0 | 0.0 | 0.0 |
| nad | 0.0 | 0.0 | 0.0 | 0.0 | 0.0 | 0.0 |
| afi | 0.0 | 0.0 | 0.0 | 0.0 | 0.0 | 0.0 |
| pam | 0.0 | 0.0 | 0.0 | 0.0 | 0.0 | 0.0 |
| zma | 0.0 | 0.0 | 0.0 | 0.0 | 0.0 | 0.0 |
| mac | 0.0 | 0.0 | 0.0 | 0.0 | 0.0 | 0.0 |
| mgi | 0.0 | 0.0 | 0.0 | 0.0 | 0.0 | 0.0 |
| sbi | 0.0 | 0.0 | 0.0 | 0.0 | 0.0 | 0.0 |
| zma | 0.0 | 0.0 | 0.0 | 0.0 | 0.0 | 0.0 |
| pvi | 0.0 | 0.0 | 0.0 | 0.0 | 0.0 | 0.0 |
| sit | 0.0 | 0.0 | 0.0 | 0.0 | 0.0 | 0.0 |
| hvu | 0.0 | 0.0 | 0.0 | 0.0 | None | 0.0 |
| tae | 0.0 | 0.0 | 0.0 | 0.0 | None | 0.0 |
| osa | 0.0 | 0.0 | 0.0 | 0.0 | None | 0.0 |
| vvi | 299.3837826371092 | None | None | 13.686115777696422 | 1.7107644722120527 | 13.686115777696422 |
| csi | 309.0580166254825 | None | None | None | None | 18.38789114934221 |
| cpa | None | None | 29.867873200279988 | None | None | 0.0 |
| gar | 9.673944385429916 | 145.4212285035594 | None | 0.31206272211064245 | None | 0.6241254442212849 |
| ath | 35.72563696346869 | None | None | 3.695755547945037 | None | 2.463837031963358 |
| cma | 0.0 | 0.0 | 0.0 | 0.0 | 0.0 | 0.0 |
| mtr | 0.0 | 0.0 | 0.0 | 0.0 | 0.0 | 0.0 |
| pvu | 0.0 | 0.0 | 0.0 | 0.0 | 0.0 | 0.0 |
| sla | 30.910823068033928 | None | None | None | None | 2.3777556206179944 |
| lsa | 76.44262142257854 | None | None | None | 0.9322270905192505 | 5.593362543115503 |
| mgu | 27.392396073756565 | None | None | None | None | 4.565399345626094 |
| nta | 89.01854374242538 | None | None | 2.502529342156537 | 2.1450251504198885 | 5.005058684313074 |
| phy | 51.32066175880134 | None | None | 0.3018862456400079 | None | 5.1320661758801345 |
| can | 31.44385134353288 | None | None | 0.3144385134353288 | None | 2.5155081074826304 |
| sly | None | None | None | None | None | 0.0 |
| stu | None | None | 4.959348837497637 | None | None | 0.0 |(B)
### Chart
| Category | UUAGAUUCACGCACAAACUCG | UUAGAUUCACGCAGAAACUCG | CUAGAUUCACGCACAAACUCG | UUAGAUUCACGCACAAACUCU | UUAGAUUCACGCACAAACUCA | Rest |
|---|---|---|---|---|---|---|
| cco | 0.0 | 0.0 | 0.0 | 0.0 | 0.0 | 0.0 |
| ppa | 0.0 | 0.0 | 0.0 | 0.0 | 0.0 | 0.0 |
| sma | 0.0 | 0.0 | 0.0 | 0.0 | 0.0 | 0.0 |
| mqu | 0.0 | 0.0 | 0.0 | 0.0 | 0.0 | 0.0 |
| cru | 0.0 | 0.0 | 0.0 | 0.0 | 0.0 | 0.0 |
| gbi | 0.0 | 0.0 | 0.0 | 0.0 | 0.0 | 0.0 |
| pab | 0.0 | 0.0 | 0.0 | 0.0 | 0.0 | 0.0 |
| nad | 0.0 | 0.0 | 0.0 | 0.0 | 0.0 | 0.0 |
| afi | 0.0 | 0.0 | 0.0 | 0.0 | 0.0 | 0.0 |
| pam | 0.0 | 0.0 | 0.0 | 0.0 | 0.0 | 0.0 |
| zma | 0.0 | 0.0 | 0.0 | 0.0 | 0.0 | 0.0 |
| mac | 0.0 | 0.0 | 0.0 | 0.0 | 0.0 | 0.0 |
| mgi | 0.0 | 0.0 | 0.0 | 0.0 | 0.0 | 0.0 |
| sbi | 0.0 | 0.0 | 0.0 | 0.0 | 0.0 | 0.0 |
| zma | 0.0 | 0.0 | 0.0 | 0.0 | 0.0 | 0.0 |
| pvi | 0.0 | 0.0 | 0.0 | 0.0 | 0.0 | 0.0 |
| sit | 0.0 | 0.0 | 0.0 | 0.0 | 0.0 | 0.0 |
| hvu | 0.0 | 0.0 | 0.0 | 0.0 | None | 0.0 |
| tae | 0.0 | 0.0 | 0.0 | 0.0 | None | 0.0 |
| osa | 0.0 | 0.0 | 0.0 | 0.0 | None | 0.0 |
| vvi | 299.3837826371092 | None | None | 13.686115777696422 | 1.7107644722120527 | 13.686115777696422 |
| csi | 309.0580166254825 | None | None | None | None | 18.38789114934221 |
| cpa | None | None | 29.867873200279988 | None | None | 0.0 |
| gar | 9.673944385429916 | 145.4212285035594 | None | 0.31206272211064245 | None | 0.6241254442212849 |
| ath | 35.72563696346869 | None | None | 3.695755547945037 | None | 2.463837031963358 |
| cma | 0.0 | 0.0 | 0.0 | 0.0 | 0.0 | 0.0 |
| mtr | 0.0 | 0.0 | 0.0 | 0.0 | 0.0 | 0.0 |
| pvu | 0.0 | 0.0 | 0.0 | 0.0 | 0.0 | 0.0 |
| sla | 30.910823068033928 | None | None | None | None | 2.3777556206179944 |
| lsa | 76.44262142257854 | None | None | None | 0.9322270905192505 | 5.593362543115503 |
| mgu | 27.392396073756565 | None | None | None | None | 4.565399345626094 |
| nta | 89.01854374242538 | None | None | 2.502529342156537 | 2.1450251504198885 | 5.005058684313074 |
| phy | 51.32066175880134 | None | None | 0.3018862456400079 | None | 5.1320661758801345 |
| can | 31.44385134353288 | None | None | 0.3144385134353288 | None | 2.5155081074826304 |
| sly | None | None | None | None | None | 0.0 |
| stu | None | None | 4.959348837497637 | None | None | 0.0 |Reads per million
Figure S8. Abundance and sequence diversity of miR403 members across plant families in reproductive tissues. a abundance of miR403. Color bars represent most abundant forms of miR403 in reproductive tissues. b Percentage abundance of miR403 across plant families.
