# Supplementary material for: Diversity, expression and mRNA targeting abilities of Argonaute-targeting miRNAs among selected vascular plants
Source: BMC Genomics. 2014 Dec 2;15(1):1049. doi: 10.1186/1471-2164-15-1049 (PMC4300679; doi:10.1186/1471-2164-15-1049)
Supplement: Supplementary file 10 — Additional file 10: Figure S9: Ago2 targeting abilities of miR403 across plants. (PPTX 239 KB) [file 12864_2014_6764_MOESM10_ESM.pptx]

## Slide 1
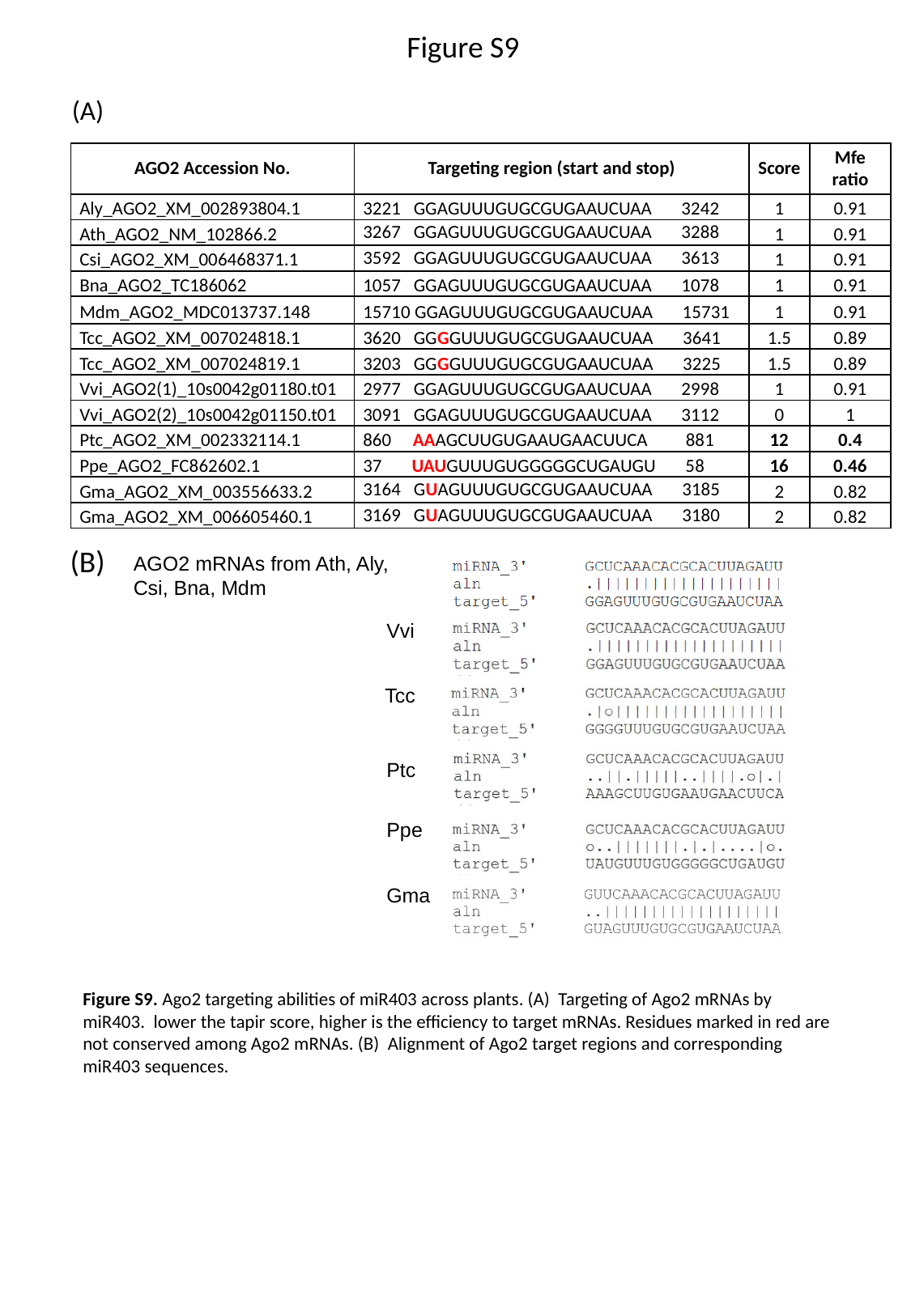

Figure S9
(A)
| AGO2 Accession No. | Targeting region (start and stop) | Score | Mfe ratio |
| --- | --- | --- | --- |
| Aly\_AGO2\_XM\_002893804.1 | 3221 GGAGUUUGUGCGUGAAUCUAA 3242 | 1 | 0.91 |
| Ath\_AGO2\_NM\_102866.2 | 3267 GGAGUUUGUGCGUGAAUCUAA 3288 | 1 | 0.91 |
| Csi\_AGO2\_XM\_006468371.1 | 3592 GGAGUUUGUGCGUGAAUCUAA 3613 | 1 | 0.91 |
| Bna\_AGO2\_TC186062 | 1057 GGAGUUUGUGCGUGAAUCUAA 1078 | 1 | 0.91 |
| Mdm\_AGO2\_MDC013737.148 | 15710 GGAGUUUGUGCGUGAAUCUAA 15731 | 1 | 0.91 |
| Tcc\_AGO2\_XM\_007024818.1 | 3620 GGGGUUUGUGCGUGAAUCUAA 3641 | 1.5 | 0.89 |
| Tcc\_AGO2\_XM\_007024819.1 | 3203 GGGGUUUGUGCGUGAAUCUAA 3225 | 1.5 | 0.89 |
| Vvi\_AGO2(1)\_10s0042g01180.t01 | 2977 GGAGUUUGUGCGUGAAUCUAA 2998 | 1 | 0.91 |
| Vvi\_AGO2(2)\_10s0042g01150.t01 | 3091 GGAGUUUGUGCGUGAAUCUAA 3112 | 0 | 1 |
| Ptc\_AGO2\_XM\_002332114.1 | 860 AAAGCUUGUGAAUGAACUUCA 881 | 12 | 0.4 |
| Ppe\_AGO2\_FC862602.1 | 37 UAUGUUUGUGGGGGCUGAUGU 58 | 16 | 0.46 |
| Gma\_AGO2\_XM\_003556633.2 | 3164 GUAGUUUGUGCGUGAAUCUAA 3185 | 2 | 0.82 |
| Gma\_AGO2\_XM\_006605460.1 | 3169 GUAGUUUGUGCGUGAAUCUAA 3180 | 2 | 0.82 |
(B)
AGO2 mRNAs from Ath, Aly, Csi, Bna, Mdm
Vvi
Tcc
Ptc
Ppe
Gma
Figure S9. Ago2 targeting abilities of miR403 across plants. (A) Targeting of Ago2 mRNAs by miR403. lower the tapir score, higher is the efficiency to target mRNAs. Residues marked in red are not conserved among Ago2 mRNAs. (B) Alignment of Ago2 target regions and corresponding miR403 sequences.
