# Supplementary material for: Diversity, expression and mRNA targeting abilities of Argonaute-targeting miRNAs among selected vascular plants
Source: BMC Genomics. 2014 Dec 2;15(1):1049. doi: 10.1186/1471-2164-15-1049 (PMC4300679; doi:10.1186/1471-2164-15-1049)
Supplement: Supplementary file 11 — Additional file 11: Figure S10: Ago3 targeting by miR403. (PPTX 51 KB) [file 12864_2014_6764_MOESM11_ESM.pptx]

## Slide 1
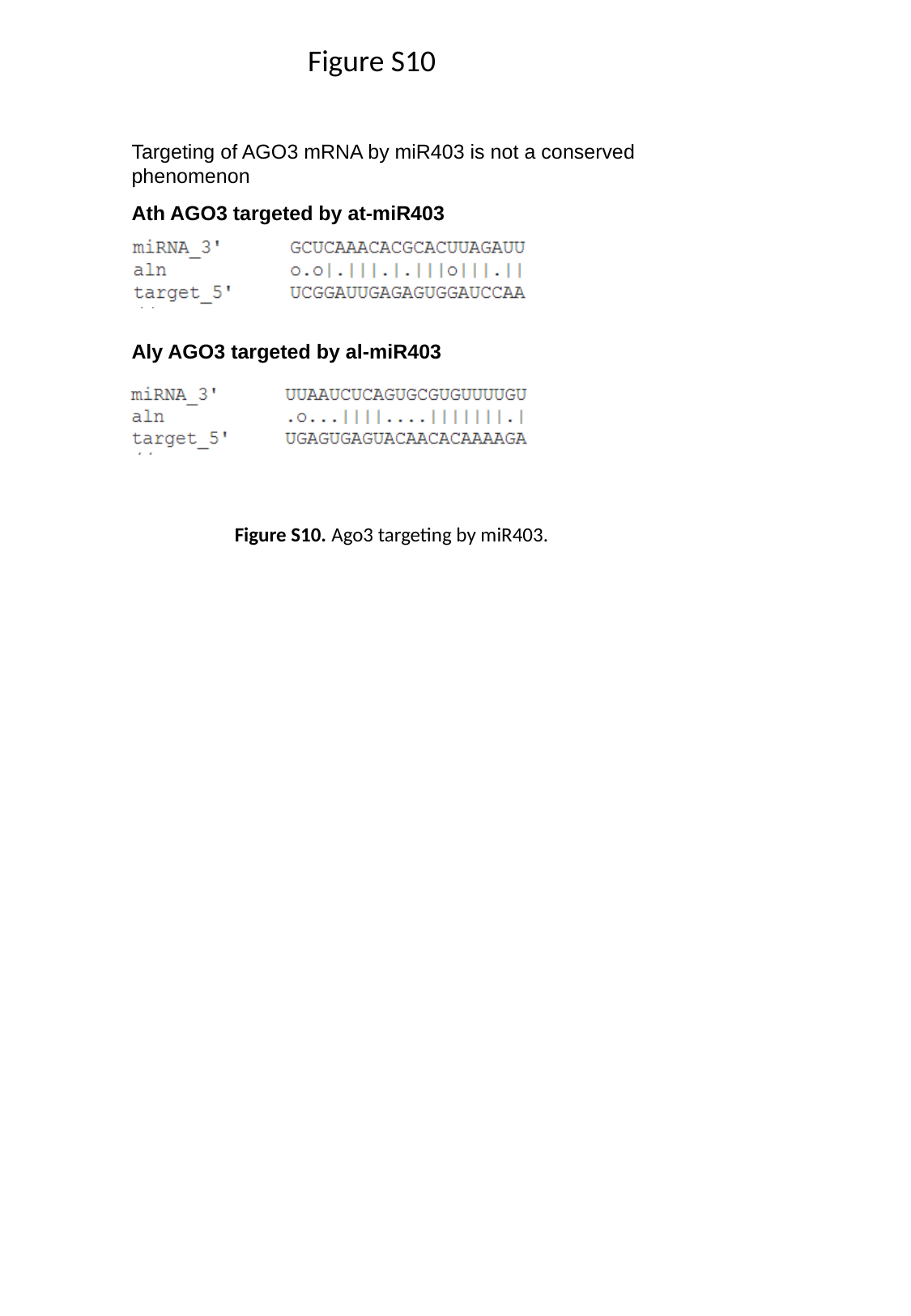

Figure S10
Targeting of AGO3 mRNA by miR403 is not a conserved phenomenon
Ath AGO3 targeted by at-miR403
Aly AGO3 targeted by al-miR403
Figure S10. Ago3 targeting by miR403.
